# Efficacy of an online self-help programme with automated or individualised psychological support versus treatment as usual for caregivers of people with depression: a randomised, controlled, open-label, superiority trial

**DOI:** 10.1016/j.lanepe.2025.101560

**Published:** 2025-12-11

**Authors:** Elisabeth Schramm, Nadine Zehender, Christoph Breuninger, Ulrich Hegerl, Anne Elsner, Andy Maun, Marina Schmölz, Christiane Roick, Jörg Sahlmann, Marlon Grodd, Katharina Domschke, Moritz Elsaesser, Erika Graf

**Affiliations:** a Department of Psychiatry and Psychotherapy, Medical Center – University of Freiburg, Faculty of Medicine, University of Freiburg, Freiburg, Germany; b Department of Psychology, Laboratory for Biological Psychology, Clinical Psychology and Psychotherapy, University of Freiburg, Freiburg, Germany; c Department of Psychiatry, Psychosomatic Medicine and Psychotherapy, University Hospital Frankfurt, Goethe University Frankfurt (Distinguished Professorship funded by Dr. Senckenbergische Stiftung), Frankfurt am Main, Germany; d German Depression Foundation, Leipzig, Germany; e Institute of General Practice / Family Medicine, Faculty of Medicine and Medical Center – University of Freiburg, Freiburg, Germany; f AOK Federal Association, Berlin, Germany; g Institute of Medical Biometry and Statistics, Medical Center – University of Freiburg, Faculty of Medicine, University of Freiburg, Freiburg, Germany

**Keywords:** Online self-help programme, Caregivers, Depression, Automated support, Individualised support, Randomised controlled trial

## Abstract

**Background:**

Informal caregivers of depressed individuals often report extensive psychological distress with detrimental consequences for their own health and for the course of the depressed person's disease. An online self-help programme was developed involving focus groups of caregivers, affected individuals and clinical experts.

**Methods:**

In this randomised, controlled, open-label superiority trial with three parallel groups (prospective registration: DRKS00025241), stratified randomisation allocated caregivers (≥18 years) of depressed individuals in a 2:2:1 ratio to receiving the programme with either individualised (IND, three weekly e-mails by trained psychologists) or automated (AUT) support messages, or to treatment as usual (TAU; information material, no online programme, no messages). The primary outcome was the change from baseline to four weeks after randomisation in the Kessler Psychological Distress Scale (K-10).

**Findings:**

The trial was conducted between 01 March 2020 and 29 February 2024. In 1640 (IND: *n* = 651; AUT: *n* = 659; TAU: *n* = 330) caregivers (mean[SD] age *m*_*age*_ = 42·8 [12·89] years; *n* = 1300 [79%] female; baseline K-10 score *m*_K-10_ = 23·4 [6·09]), both IND and AUT reduced psychosocial distress significantly more than TAU at four weeks after randomisation (adjusted difference *m*_*IND/TAU*_ [95%-CI] = –1·45 [–2·19,–0·72], *p* = 0·0001; *m*_*AUT/TAU*_ [95%-CI] = –0·89 [–1·63,–0·14], *p* = 0·0205; dropout rate: 34%, *n* = 562). No study-related harms were reported.

**Interpretation:**

Psychological online support for caregivers of depressed individuals with either individualised or automated messages is effective in decreasing their psychosocial distress and could be offered as part of integrated services.

**Funding:**

German Innovation Fund (Federal Joint Committee, 01VSF19054).

**Research in context**

**Evidence before this study**

We conducted a systematic literature search of MEDLINE via Ovid and PsycINFO databases using the search terms (and their synonyms/truncations) “caregiver”, “caretaker”, “carer”, “significant other”, “relative”, “automated support”, “individualiz(s)ed support”, “online program(me)”, “online intervention”, “psychoeducation”, “self-help”, and “depression”. There were no restrictions regarding the publication date or language. Additionally, forward and backward citation searches of relevant articles were performed. The literature search was last updated on August 7, 2025. We based our research on the finding of several studies that psychoeducational face-to-face programmes reduced the burden on relatives of people with depression. A systematic review and meta-analysis of Katsuki and colleagues in 2022 investigating face-to-face psychoeducation for families of depressed individuals showed positive results both in terms of the families’ strain and the depressive symptoms of the affected person. There are no empirically evaluated programmes available as online interventions as shown in the only systematic review on this topic by Meyer and colleagues in 2018 despite advantages such as anonymity, easy accessibility, flexibility, and cost-effectiveness. In particular, the differential effects of automated versus individualised psychological support embedded within this setting has not yet been examined in a randomised controlled trial.

**Added value of this study**

This is the first randomised controlled trial evaluating the efficacy of an online self-help programme for informal caregivers of people with depression, directly comparing automated versus human psychological support within the programme. In a large sample of 1640 informal caregivers of depressed individuals, the online self-help with either automated or individualised messages reduced the psychosocial distress in the caregivers statistically significantly more compared to receiving information material only (treatment as usual). Caregivers in both active interventions also reported considerable improvement in psychological symptoms, and in their family interaction behaviour. However, there were no significant differences between both support conditions. In the group of depressed significant others of the caregivers, there was a decrease in depressive symptoms over the measurement period, although no significant group differences between conditions were found.

**Implications of all the available evidence**

This trial provides evidence of the efficacy, acceptance, and safety of an online self-help programme for informal caregivers of people with depression. Our results demonstrate substantial reductions of both psychological distress and subjective symptom burden in caregivers, with potential secondary benefits for depressed individuals. As such it has the potential to be integrated as a low-threshold and flexible support tool in routine healthcare services. Since automated support was nearly as effective as individualised guidance, implementation of digital caregiver support programmes can address workforce shortages and logistical barriers, extending preventive mental health support to underserved and vulnerable populations. Future research should explore longer-term outcomes, impact on recovery and relapse rates of the people with depression, and implementation into routine health care systems.

## Introduction

Depressive disorders are one of the leading factors contributing to global disability and disease burden.^1^ In a representative survey in Germany with 5000 participants, a significant number of individuals reported to be affected by depression either directly (24%) or indirectly as family members (26%) over their lifetime.^2^ While Western healthcare systems offer various treatment modalities for depressive disorders, the distress of caregivers is frequently overlooked by physicians^3^ – even though the illness-related burden has negative consequences for the carers' mental and physical health.^4^ Compared with caregivers of patients with other chronic illnesses, carers of depressed individuals show a larger strain in terms of poor quality of life, marked impairment of productivity, and increased health resource utilisation.^5^ They also have a higher risk of developing depression themselves^6^^,^^7^ with a doubled one-year prevalence compared to the general population.^8^ In addition, increased family burden raises the risk of relapse and chronicity of the depressed individual.^9^, ^10^, ^11^

The important role of caregiving family members – as a vulnerable group as well as a potential supportive resource – is emphasised in national and international clinical practice guidelines for the treatment of depression (e.g. NICE,^12^ RANZCP,^12^ NVL^13^), which recommend psychoeducational support for carers and significant others. Psychoeducational programmes reduce the family burden and are linked to decreased depressive symptoms and relapse rates of the affected person.^14^, ^15^, ^16^ However, there is an undersupply of psychoeducational offers for families of patients with depression.^17^ To close this gap in the provision of services, an interactive online self-help programme for informal caregivers of depressed adult individuals was developed. To our knowledge, this is the first evidence-based online intervention currently available for non-professional caregivers of depressed patients.^18^

This study aimed to evaluate the efficacy of our online programme, supplemented by either automated (AUT) or individualised (IND) support messages, compared to a treatment as usual condition (TAU; information material for significant others of depressed individuals, no online programme, no messages). The TAU condition is based on the recommendation of the national treatment guidelines for depression (NVL^13^) to educate significant others about their relatives‘ illness. Our primary hypothesis was that the online programme with either IND or AUT would reduce nonspecific mental distress in caregivers more than TAU. We further expected IND to be more effective than AUT based on the finding that human support is often reported to be more efficacious than automated guidance in text-based internet treatments as shown in a meta-analysis.^19^ Lastly, we hypothesised that both versions would reduce the caregivers’ subjective symptom burden as well as the affected persons' depressive symptoms and improve interactive behaviour in the family.

## Methods

### Study design

This is a randomised, controlled, open-label superiority trial with online randomisation into three parallel groups conducted between 01 March 2020 and 29 February 2024. Caregivers were recruited in practices of general practitioners, psychiatrists, and psychotherapists as well as in out- and inpatient clinics throughout Germany from 06 May 2021 to 31 January 2023. Another recruitment strategy included activities in a network of 85 regional “Alliances against Depression” coordinated by the German Depression Foundation, their website, social media channels, and magazines (more details on recruitment sources are depicted in the supplement). The study was conducted via an online platform accessible through all common internet browsers without additional phone or face-to-face contact. The Innovation Fund took over the monitoring function of a Data and Safety Monitoring Board (DSMB). The study was prospectively registered in the German Clinical Trials Register (DRKS00025241) on 5 May 2021. Additionally, the study protocol was published as an open access article (https://doi.org/10.1186/s12888-022-04035-6).^20^

### Participants

**Fig. 1:**
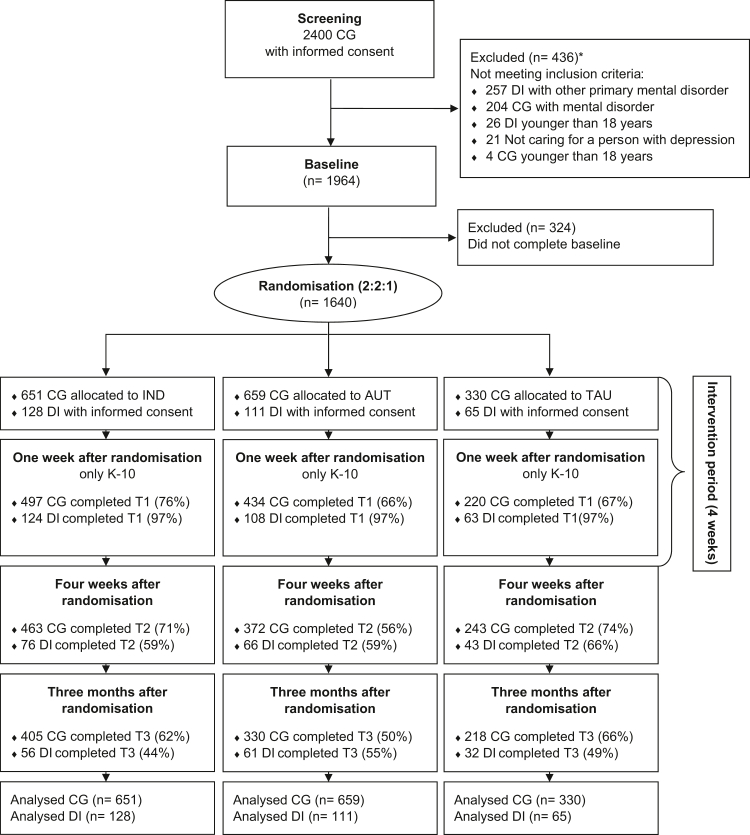
CONSORT flow diagram. CG, caregivers; DI, depressed individuals; IND, online programme with individualised support; AUT, online programme with automated support; TAU, treatment-as-usual. ∗ Multiple items can apply.

Eligibility required a primary diagnosis of unipolar depressive disorder or primarily depressive symptoms in the depressed person, and absence of current mental disorders in the caregivers (via self-report item), because otherwise priority should be on their own disorder first. With regard to the Kessler Psychological Distress Scale (K-10), there was no cut-off for inclusion in the study. Caregivers were offered to invite their depressed person to take part in the assessments. However, caregivers could also participate without the depressed individual and were the primary target of the study. Caregivers and depressed persons had to be at least 18 years of age and possess sufficient German language skills. A total of *n* = 2400 caregivers were screened, of whom *n* = 436 individuals were excluded for not meeting the eligibility criteria and *n* = 324 were excluded for not completing the baseline assessment, so that *n* = 1640 subjects were randomised (see Fig. 1). Participants reported their gender, biological sex was not separately recorded. Race and ethnicity data were not collected.

### Randomisation and masking

Eligible caregivers were automatically randomised via the study platform to the intervention and control groups IND, AUT and TAU with a 2:2:1 ratio. Assignment was based on computer-generated lists and stratified by psychosocial distress (K-10; 10–22, 23–50 points), age (18–40, 41–65, ≥66 years), gender (female, male; caregivers of diverse gender were alternatingly assigned with females and males), and relation with depressed person (parent, child, partner, other), with a concealed constant block size of 5. These stratification factors were selected due to their expected prognostic relevance for the primary K-10 outcome scale. In particular, the K-10 cut-off value was chosen based on its ability to differentiate healthy persons from persons at risk of a mental disorder in the German validation study.^21^ Due to the nature of interventions, neither the caregivers nor the study team were blinded.

### Procedures

Following online written informed consent and eligibility screening, caregivers completed the pre-intervention questionnaires (baseline) and were immediately randomised to start one of three four-week intervention conditions. One week after randomisation, they completed an interim questionnaire assessing psychological distress (K-10) and again after the four-week intervention period (primary outcome), in addition to other psychological questionnaires and data collected on programme acceptance, adherence, usage, and adverse events. Three months after randomisation, caregivers reported again on the psychological questionnaires and adverse events.

The caregivers’ depressed significant others were also invited to participate by completing the Patient Health Questionnaire (PHQ-9)^22^ at baseline, four weeks after randomisation, and three-month follow-up, though no intervention was provided directly to them. If the depressed individual participated in the study, their informed consent was required prior to participation.

### Interventions

**Fig. 2:**
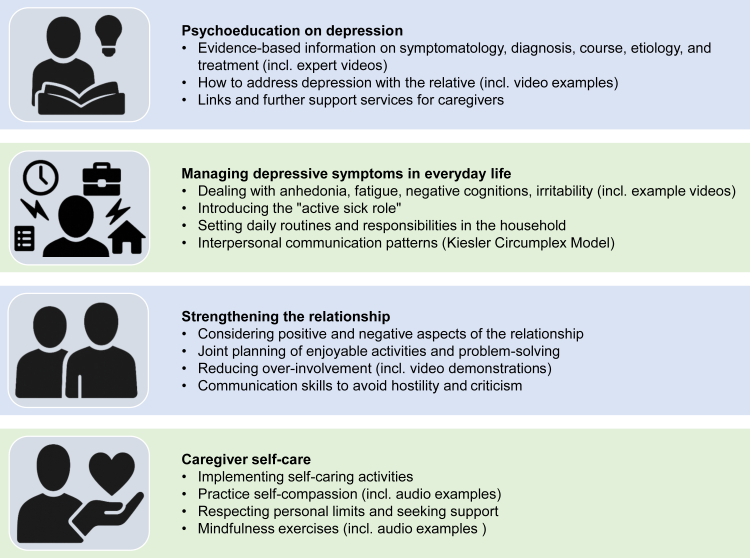
Structure and modules of the online self-help programme.

The online self-help programme consists of four independent interactive modules on psychoeducation, dealing with the patient's depressive symptoms, strengthening the relationship, and self-care. The content of the modules was developed in our work group (based on long-term experience in the treatment of people with depression and their significant others) as a joint consensus project involving focus groups of caregivers, affected individuals and clinical experts. The content of all modules is based on scientific evidence from research on expressed emotion (criticism and over-involvement in the communication between the relatives and the patient), communication and interpersonal conflicts in families of depressed adults, and from depression psychoeducation. In the different modules, caregivers have access to instructions, video examples and exercises (see Fig. 2). The caregivers were free to choose the sequence of the modules. Each module takes approximately 1·5 to 2·5 h to complete, including homework exercises.

Caregivers were randomly assigned to one of the following conditions:

*Online self-help programme with individual support (IND)* including personal messages to the caregivers three times a week by trained psychologists under regular supervision from experienced psychotherapists. The psychologists provided individual guidance on relevant content, additional information tailored to the caregivers' specific situation, personalised strategies to deal with the situation, answers to their questions, and motivational support. Caregivers received messages in a separate messaging section of the online programme in which they could also respond to the psychologists. Each support psychologist was provided with an individual account, through which they received information about the caregivers they supported (e.g., age, gender, relationship to the depressed individual, programme progress) and send messages. A typical sequence of individualised support messages in the IND condition is illustrated in the supplement.

*Online self-help programme with automated support (AUT)* including fully automated, personalised messages to the caregivers three times a week. The messages provided encouragement, instructions for the online programme and feedback on completed modules, tailored to each caregiver's activities and progress in the online programme (e.g., information related to previously completed content, weekly progress summaries, and feedback in cases of high or low usage). Caregivers could not respond to these messages. A typical sequence of automated support messages in the AUT condition is illustrated in the supplement.

*Treatment as usual (TAU) with information material* consisting of a digital version of a two-page information leaflet on depressive symptoms influencing everyday life, supporting a depressed person and on practicing self-care. After the three-month study period, this group was offered free access to the automated online programme.

Caregivers in all groups were free to use medical or psychological services as usual, though specific support services for caregivers are rare and often not covered by the health insurance systems.

### Involvement of people with lived experience

In the initial phase, people with lived experiences were engaged in the development of the online programme and in addition, provided feedback on optimising the message support. Caregivers’ associations were also involved in the planning of the recruitment. Within focus groups on dissemination, caregivers contributed to interpreting and contextualising the results, as well as planning further dissemination steps. Some of the authors had lived experience as caregivers of a person with depression.

### Outcomes

Primary outcome was the change from baseline to four weeks after randomisation in the Kessler Psychological Distress Scale (K-10),^21^ which assesses non-specific psychological distress over the past 30 days using 10 Items and a five-point Likert scale (1 = none of the time, 5 = all of the time). In the present study, the K-10 demonstrated good internal consistency (Cronbach's α = 0·87). The K-10 has demonstrated strong convergent validity with established measures of psychological burden, including the Global Severity Index (GSI; r = 0·71).^23^

Furthermore, several secondary outcome measures were assessed (for a comprehensive overview of all outcome measures, see supplement). The psychological symptoms of caregivers were assessed using the German version of the 9-item short version of the Symptom Checklist (SCL-K-9),^24^ which measures psychological symptoms on a five-point Likert scale (0 = not at all, 4 = very severe) and showed good internal consistency (Cronbach's α = 0·82). The level of expressed emotion is measured by the German version of the Family Questionnaire (FQ; *Familienfragebogen*, FFB),^25^ a 20-item self-report measure consisting of two subscales, ‘criticism’ and ‘emotional over-involvement’. This scale was used because high expressed emotion has been identified as a risk factor for the exacerbation and course of the patients' mental illness. The FQ showed good internal consistency (Cronbach's α = 0·88). On a four-point Likert scale, ranging from “never” to “very often”, caregivers of patients evaluate the frequency of specific reactions towards the patient. Depressive symptoms were measured with the Patient Health Questionnaire (PHQ-9).^22^^,^^26^ Subjects indicate for each of the nine items on a four-point scale, ranging from 0 (never) to 3 (nearly every day), whether the symptom has bothered them during the previous 2 weeks. The PHQ-9 demonstrated acceptable internal consistency (Cronbach's α = 0·80).

Furthermore, usage data of the online programme was collected for each caregiver, including progress in the modules, weekly time spent working with the programme, and the number of messages to the psychologist in the IND condition. Adherence was defined as using the programme for more than 90 min during the 4-week study period and completing more than 25% of the module content.

Further questionnaires, the Involvement Evaluation Questionnaire - European Version (IEQ-EU), the Depression Literacy Questionnaire (D-Lit), and the World Health Organization Five Well-Being Index (WHO-5), which were collected as additional secondary outcomes within the study project, will be published separately in combination with and related to qualitative data.

### Statistical analysis

A prospective Statistical Analysis Plan (SAP; see supplement) was developed prior to the statistical analyses and approved by the steering committee on 8 May 2023. The primary intention-to-treat analysis included all randomised caregivers in their assigned arms. We replaced missing values via multiple imputation using baseline and post-baseline data, including usage markers of the online support for IND and AUT arms. A linear regression estimated the effects of allocation on the change in K-10 score from baseline to four weeks after randomisation, adjusting for baseline K-10 score, age, caregiver relationship, and gender. A sequential closed testing procedure was implemented using a pre-specified order of pairwise comparisons to control the family-wise type I error rate at 5%. Confirmatory testing at the 5% level proceeded only until the first non-significant result; subsequent analyses were reported descriptively. To compare interventions, we tested the difference in mean changes from baseline using two-sided 95% confidence intervals from the linear regression model. Interventions were compared in the following pre-specified order: IND versus TAU, AUT versus TAU, IND versus AUT. While we primarily hypothesised and tested for superiority of IND over AUT regarding the third comparison, a secondary descriptive analysis explored the non-inferiority of AUT to IND. Non-inferiority was inferred if the lower limit of the two-sided 95% confidence interval for the mean K-10 change difference (IND minus AUT) exceeded −0·62 points.

Power calculations assumed a standard deviation of 6·2 K-10 points, so Cohen's *d* values of 0·5, 0·3, 0·2, 0·1 corresponded to differences between group means of 3·10, 1·86, 1·24, 0·62 K-10 score points. Based on the sample size calculation, we initially planned for 1000 non-missing primary outcomes (*n* = 400:400:200, IND:AUT:TAU) at four weeks after randomisation, assuming an 80% retention rate and therefore targeting 1250 randomisations. This target was subsequently revised to 1640 upon identifying an actual retention rate of 61%, to ensure approximately 1000 non-missing outcomes. With 1000 (*n* = 400:400:200, IND:AUT:TAU) non-missing primary outcomes at four weeks after randomisation, the comparisons of the online programmes IND and AUT with TAU were powered at 1-β≈99% for a medium effect size (*d* = 0·5) and 93% for *d* = 0·3, while the IND versus AUT comparison had 81% power to detect a small effect (*d* = 0·2; further details are provided in the supplement). Sensitivity analyses assessed the missing data mechanism and the impact of adherence. In a conservative scenario, we assumed that missing outcomes in the IND and AUT arms followed the distribution observed in the TAU group.

Secondary outcome scales were evaluated in pre-specified linear mixed models for repeated measures with a compound symmetry covariance structure, in line with the SAP where they were also referred to as longitudinal ANCOVA (see supplement for details). Outcomes per intervention were estimated using least squares means. We computed descriptive *post-hoc* effect sizes *d* by dividing the adjusted mean difference between arms by the empirical pooled standard deviation of observed outcomes, weighted by group size at the respective time point. A *post-hoc* Welch two-sample t-test compared the time spent on programme use among caregivers of the active intervention arms who engaged with the programme (i.e., who used it >0 min). Cronbach's alpha was calculated *post-hoc* for baseline K-10, SCL-K-9 and FQ questionnaires in all randomised caregivers, and for baseline PHQ-9 scores of participating depressed persons. Statistical computing was performed using SAS 9·4 and R (4·3·1 for main analyses; 4·5·1 for *post-hoc* analyses).

### Ethics approval

This study obtained approval from the independent Ethics Committees of the University of Freiburg (11 February 2021, reference number 20–1276; approval for an amendment with the inclusion of process and qualitative analyses on 16 November 2021, reference 20–1276_2; approval for an amendment to increase the sample size on 11 August 2022, reference number 20–1276_3).

### Role of the funding source

The funding body had no role in the design and conduct of the study; collection, management, analysis, and interpretation of the data; preparation, review, or approval of the manuscript; or decision to submit the manuscript for publication.

## Results

### Study population

The participating caregivers had a mean age (SD) of *m*_*age*_ = 42·8 (12·89) years, *n* = 1300 (79%) were female. The depressed persons had a mean (SD) age of *m*_*age*_ = 40·6 (15·57) years and were predominantly male (*n* = 951, 58%). Most participating caregivers had a depressed partner (*n* = 973, 59%), followed by an adult child (*n* = 299, 18%), a parent (*n* = 187, 11%), or another significant other (*n* = 181, 11%). The mean (SD) K-10 score of *m* = 23·4 (6·09) of the caregivers indicates a high level of psychosocial distress associated with a significant risk of developing mental health symptoms.^21^ Characteristics were evenly distributed across the randomised groups. Table 1 provides sample characteristics delineated by study condition.

**Table 1:** Baseline characteristics.

	IND	AUT	TAU	Total
**Caregivers**				
Randomised, No.	651	659	330	1640
Gender, No. (%)				
Female	517 (79%)	522 (79%)	261 (79%)	1300 (79%)
Male	134 (21%)	136 (21%)	68 (21%)	338 (21%)
Diverse	0 (0%)	1 (0%)	1 (0%)	2 (0%)
Age, y, mean (SD)	42·7 (12·81)	42·9 (12·83)	42·9 (13·21)	42·8 (12·89)
Age, No. (%)				
18–40	293 (45%)	298 (45%)	151 (46%)	742 (45%)
41–65	338 (52%)	338 (51%)	167 (51%)	843 (51%)
>65	20 (3%)	23 (3%)	12 (4%)	55 (3%)
Education, No. (%)				
None	0 (0%)	1 (0%)	0 (0%)	1 (0%)
Lower secondary	21 (3%)	14 (2%)	10 (3%)	45 (3%)
Medium secondary (10 yrs)	125 (19%)	147 (22%)	66 (20%)	338 (21%)
Upper secondary (11–13 yrs)	458 (70%)	444 (67%)	229 (69%)	1131 (69%)
Other	47 (7%)	53 (8%)	25 (8%)	125 (8%)
Employment status, No. (%)				
Student	189 (29%)	195 (30%)	93 (28%)	477 (29%)
Employed	350 (54%)	338 (51%)	160 (48%)	848 (52%)
Not employed	22 (3%)	37 (6%)	19 (6%)	78 (5%)
Retired	24 (4%)	27 (4%)	16 (5%)	67 (4%)
Other	66 (10%)	62 (9%)	42 (13%)	170 (10%)
K-10 score, mean (SD)	23·5 (5·95)	23·5 (6·26)	23·2 (6·03)	23·4 (6·09)
SCL-K-9 score, mean (SD)	12·2 (6·10)	12·3 (6·55)	12·2 (6·48)	12·2 (6·36)
FQ score, mean (SD)	21·5 (4·61)	21·6 (4·70)	21·4 (4·64)	21·5 (4·65)
Common household with the depressed individual, No. (%)				
No	246 (38%)	245 (37%)	130 (39%)	621 (38%)
Yes	405 (62%)	414 (63%)	200 (61%)	1019 (62%)
Relation to the depressed individual, No. (%)				
Partner	389 (60%)	390 (59%)	194 (59%)	973 (59%)
Parent	121 (19%)	119 (18%)	59 (18%)	299 (18%)
Adult child	73 (11%)	76 (12%)	38 (12%)	187 (11%)
Other	68 (10%)	74 (11%)	39 (12%)	181 (11%)
Gender of the depressed individual, No. (%)				
Female	277 (43%)	277 (42%)	127 (38%)	681 (42%)
Male	370 (57%)	379 (58%)	202 (61%)	951 (58%)
Diverse	4 (1%)	3 (0%)	1 (0%)	8 (0%)
Age of the depressed individual, y, mean (SD)	40·8 (15·49)	40·6 (15·47)	40·4 (15·95)	40·6 (15·57)
**Participating depressed individuals**				
Total No.	128	111	65	304
Gender, No. (%)				
Female	68 (53%)	52 (47%)	29 (45%)	149 (49%)
Male	59 (46%)	59 (53%)	36 (55%)	154 (51%)
Diverse	1 (1%)	0 (0%)	0 (0%)	1 (0%)
Age, y, mean (SD)	38·6 (14·04)	37·8 (12·06)	41·8 (14·32)	39·0 (13·45)
Relation to the caregiver, No. (%)				
Partner	91 (71%)	86 (77%)	55 (85%)	232 (76%)
Adult child	21 (16%)	12 (11%)	7 (11%)	40 (13%)
Parent	9 (7%)	4 (4%)	2 (3%)	15 (5%)
Other	7 (5%)	9 (8%)	1 (2%)	17 (6%)
PHQ-9 score, mean ± SD	16·2 (5·33)	15·8 (5·24)	16·5 (5·17)	16·1 (5·25)

K-10, Kessler Psychological Distress Scale; SCL-K-9, Symptom Checklist-Short Version; FQ, Family Questionnaire; PHQ-9, Patient Health Questionnaire; IND, Online self-help programme with individual support; AUT, Online self-help programme with automated support; TAU, Treatment-as-usual.

### Programme usage

In the IND condition, caregivers who engaged with the programme (i.e., >0 min of programme use) spent an average (SD) of 287·6 min (212·40; range 14–1207) in total engaging with the programme and were active on an average (SD) of 11·7 days (6·45). In comparison, caregivers in the AUT condition spent an average (SD) of 206·8 min (179·91; range 20–1859) in the programme, with activity on an average (SD) of 8·1 days (5·40). Thus, those in the IND arm (excluding non-starters) spent on average 80·9 min more than those in the AUT arm (95%-CI: [59·5102·2], *p* < 0·0001). A total of 27 caregivers (4%) in IND and 38 caregivers (6%) in AUT did not use the programme at all (i.e., 0 min of programme use). The frequency of user-initiated messages in the IND condition varied widely among the caregivers. Specifically, 18% (*n* = 116 of 651) of caregivers did not send any messages to the support psychologist, while the remaining 82% (*n* = 535 of 651) sent an average (SD) of *m* = 5·1 messages (3·12; range 1–22) over the intervention period.

Regarding the average progress in the modules, the distribution was as follows: Managing depressive symptoms in everyday life (IND: 62·3%, AUT: 58·1%), Strengthening the relationship (IND: 57·2%, AUT: 46·7%), Caregiver self-care (IND: 64·5%, AUT: 57·7%), and Psychoeducation on depression (IND: 61·3%, AUT: 51·9%).

According to the adherence definition, 59% (*n* = 381 of 651) of caregivers in the IND group and 47% (*n* = 308 of 659) in the AUT group were classified as adherent. In the TAU group, caregivers were considered adherent by default, as no online programme was provided. Looking at the individual criteria, 81% (*n* = 530 of 651) of caregivers in the IND group used the program for more than 90 min, compared with 71% (*n* = 471 of 659) in the AUT group. Furthermore, 59% (*n* = 384 of 651) of caregivers in the IND group, versus 47% (*n* = 312 of 659) in the AUT group, completed more than 25% of the modules.

### Primary outcome scale K-10

Starting from a pooled mean (SD) of the K-10 score at baseline of *m* = 23·4 (6·09), the unadjusted mean scores observed four weeks after randomisation were *m*_*IND*_ = 21·0 (5·93) in 73% (*n* = 476 of 651), *m*_*AUT*_ = 21·4 (6·60) in 59% (*n* = 387 of 659) and *m*_*TAU*_ = 22·5 (6·18) in 78% (*n* = 258 of 330) of caregivers. After multiple imputation of missing values, linear regression including all randomised caregivers estimated mean reductions of *m*_*IND*_ = −2·29, *m*_*AUT*_ = −1·72, and *m*_*TAU*_ = −0·84 K-10 score points for caregivers in the IND, AUT, and TAU groups, respectively. In the pre-specified sequential comparison of the two experimental interventions IND and AUT with the control group TAU, both were statistically significantly more effective than TAU, with a mean difference, indicating a stronger decline, of *m*_*IND/TAU*_ = −1·45 (95%-CI: [−2·19,−0·72], *p* = 0·0001, *d* = −0·27) and *m*_*AUT/TAU*_ = −0·89 (95%-CI: [−1·63,−0·14], *p* = 0·0205, *d* = −0·17). The comparison between IND and AUT revealed a non-significant mean difference of *m*_*IND/AUT*_ = −0·57 (95%-CI: [−1·22, 0·08], *p* = 0·0914, *d* = −0·11; final confirmatory comparison). Sensitivity analyses gave essentially the same results. The results for the secondary descriptive non-inferiority analysis of AUT to IND are reported in the supplement. While the three groups did not statistically significantly differ after one week, both support programmes were superior over the control group TAU and appeared similarly effective three months after randomisation, in both the longitudinal model and after multiple imputation. An overview of all between-group differences across primary and secondary outcomes is presented in Table 2.

**Table 2:** Estimated Between-Group Differences in Psychological Outcomes from Baseline to One week after randomisation (K-10), four weeks after randomisation and three months after randomisation.

Measure	Condition A	*n*	Mean Change from baseline (SD)	Condition B	*n*	Mean Change from baseline (SD)	Mean Difference A-B (SD)	95%-CI	*p* value
**Non-specific psychological distress: Kessler Psychological Distress Scale (K-10)**									
One week after randomisation	IND	497	−1·03 (4·58)	TAU	220	−0·81 (3·91)	−0·22 (4·39)	−0·86 to 0·41	0·49
	AUT	434	−0·57 (4·06)	TAU	220	−0·81 (3·91)	0·23 (4·01)	−0·40 to 0·87	0·47
	IND	497	−1·03 (4·58)	AUT	434	−0·57 (4·06)	−0·46 (4·35)	−0·99 to 0·07	0·0914
Four weeks after randomisation	IND	476	−2·29 (5·33)	TAU	258	−0·84 (5·32)	−1·45 (5·33)	−2·19 to −0·72	0·0001
	AUT	387	−1·72 (5·15)	TAU	258	−0·84 (5·32)	−0·89 (5·22)	−1·63 to −0·14	0·0205
	IND	476	−2·29 (5·33)	AUT	387	−1·72 (5·15)	−0·57 (5·25)	−1·22 to 0·08	0·0855
Three months after randomisation	IND	414	−2·68 (5·57)	TAU	225	−1·50 (6·32)	−1·18 (5·84)	−2·05 to −0·30	0·0083
	AUT	343	−2·53 (6·06)	TAU	225	−1·50 (6·32)	−1·03 (6·16)	−1·92 to −0·15	0·0224
	IND	414	−2·68 (5·57)	AUT	343	−2·53 (6·06)	−0·15 (5·80)	−0·90 to 0·61	0·71
**Psychological symptoms: Symptom Checklist 9-Item Short Version (SCL-K-9)**									
Four weeks after randomisation	IND	469	−1·41 (5·04)	TAU	253	−0·41 (5·48)	−1·00 (5·20)	−1·74 to −0·26	0·0080
	AUT	377	−0·88 (5·31)	TAU	253	−0·41 (5·48)	−0·47 (5·38)	−1·24 to 0·30	0·23
	IND	469	−1·41 (5·04)	AUT	377	−0·88 (5·31)	−0·53 (5·16)	−1·18 to 0·12	0·11
Three months after randomisation	IND	406	−2·58 (5·72)	TAU	220	−1·06 (5·84)	−1·52 (5·76)	−2·39 to −0·65	0·0006
	AUT	334	−2·77 (6·17)	TAU	220	−1·06 (5·84)	−1·72 (6·04)	−2·62 to −0·81	0·0002
	IND	406	−2·58 (5·72)	AUT	334	−2·77 (6·17)	0·20 (5·93)	−0·57 to 0·96	0·62
**Level of Expressed Emotion: Family Questionnaire (FQ)**									
Four weeks after randomisation	IND	468	−2·65 (6·64)	TAU	252	−0·97 (6·44)	−1·68 (6·57)	−2·66 to −0·71	0·0007
	AUT	374	−1·88 (6·72)	TAU	252	−0·97 (6·44)	−0·91 (6·61)	−1·93 to 0·10	0·0771
	IND	468	−2·65 (6·64)	AUT	374	−1·88 (6·72)	−0·77 (6·68)	−1·63 to 0·09	0·0794
Three months after randomisation	IND	406	−4·67 (8·16)	TAU	219	−2·26 (8·68)	−2·41 (8·35)	−3·71 to −1·10	0·0003
	AUT	330	−3·66 (8·03)	TAU	219	−2·26 (8·68)	−1·40 (8·30)	−2·75 to −0·05	0·0425
	IND	406	−4·67 (8·16)	AUT	330	−3·66 (8·03)	−1·01 (8·10)	−2·16 to 0·14	0·0854
**Depressive Symptoms: Patient Health Questionnaire-9 (PHQ-9)**									
Four weeks after randomisation	IND	76	−2·72 (4·22)	TAU	43	−1·86 (3·07)	−0·86 (3·85)	−2·29 to 0·56	0·23
	AUT	65	−2·79 (4·06)	TAU	43	−1·86 (3·07)	−0·93 (3·70)	−2·39 to 0·53	0·21
	IND	76	−2·72 (4·22)	AUT	65	−2·79 (4·06)	0·07 (4·15)	−1·19 to 1·32	0·92
Three months after randomisation	IND	55	−3·84 (5·24)	TAU	33	−3·54 (4·63)	−0·29 (5·02)	−2·25 to 1·66	0·77
	AUT	60	−3·28 (4·96)	TAU	33	−3·54 (4·63)	0·26 (4·85)	−1·67 to 2·19	0·79
	IND	55	−3·84 (5·24)	AUT	60	−3·28 (4·96)	−0·56 (5·10)	−2·21 to 1·10	0·51

Adjusted mean changes from baseline in Kessler Psychological Distress Scale (K-10), Symptom Checklist-Short Version (SCL-K-9), Family Questionnaire (FQ), and Patient Health Questionnaire-9 (PHQ-9) scores at one week after randomisation, four weeks after randomisation and follow-up three months after randomisation. *n* and SD are numbers and standard deviations of observed, non-missing changes from baseline (for mean difference: pooled SD weighted by n's). Adjusted differences represent estimated mean changes between intervention arms based on regression models (least-squares means from linear model after multiple imputation for K-10, otherwise mixed model for repeated measures), reported with 95% confidence intervals (CIs, L lower, U upper) and *p* values for group differences. Negative differences indicate greater improvement for condition A. Abbreviations: IND, online programme with individualised support (Baseline *n* = 651); AUT, online programme with automated support (Baseline *n* = 659); TAU, treatment-as-usual (Baseline *n* = 330).

### Symptom checklist

Starting from a pooled mean (SD) of *m* = 12·2 (6·36) score points at baseline, both variants of the online programme showed better results at four weeks after randomisation and three-month follow-up in the SCL-K-9. At four weeks after randomisation, only the IND intervention demonstrated statistically significantly superior results compared to the control arm (*m*_*IND/TAU*_ = −1·00, 95%-CI: [−1·74,−0·26], *p* = 0·0080, *d* = −0·19). At three months after randomisation, both intervention arms showed statistically significant effects compared to TAU (*m*_*IND/TAU*_ = −1·52, 95%-CI: [−2·39,−0·65], *p* = 0·0006, *d* = −0·26; *m*_*AUT/TAU*_ = −1·72, 95%-CI: [2·62,−0·81], *p* = 0·0002, *d* = −0·28).

### Family questionnaire

The FQ showed a pooled mean (SD) baseline score of *m* = 51·5 (9·61) points at baseline. Scores improved across all three groups at four weeks after randomisation and even more so at the three-month follow-up assessment. The estimated improvements at both time points were more pronounced in the IND than in the AUT condition, and even more pronounced than in the TAU arm. Compared to the control arm, the individualised intervention was rated statistically significantly better, with improvements of *m*_*IND/TAU*_ = −1·68 (95%-CI: [−2·66,−0·71, *p* = 0·0007, *d* = −0·26) points at four weeks after randomisation and *m*_*IND/TAU*_ = −2.41 (95%-CI: [−3·71,−1·10], *p* = 0·0003, *d* = −0·29) points at follow-up. The differences for the automated version were slightly smaller, with differences of *m*_*AUT/TAU*_ = −0·91 (95%-CI: [−1·93,0·10], *p* = 0·0771, *d* = −0·14) points at four weeks after randomisation and *m*_*AUT/TAU*_ = −1·40 (95%-CI: [−2·75,−0·05]; *p* = 0·0425, *d* = −0·17) at follow-up. There were no statistically significant differences between IND and AUT at four weeks after randomisation (*m*_*IND/AUT*_ = −0·77, 95%-CI: [−1·63,0·09], *p* = 0·0794, *d* = −0·12) or follow-up (*m*_*IND/AUT*_ = −1·01, 95%-CI: [−2·16,0·14], *p* = 0·0854, *d* = −0·12).

### Patient health questionnaire of the depressed person

After four weeks, 61% (*n* = 186 of 304) and after three months 50% (*n* = 151 of 304) of the consenting depressed individuals participated in the PHQ-9 assessment. At baseline, the pooled mean (SD) PHQ-9 score was *m* = 16·1 (5·25) points. At four weeks after randomisation, both IND and AUT showed similar reductions, slightly more pronounced than in the control arm (*m*_*IND/TAU*_ = −0·86, 95%-CI: [−2·29,0·56], *p* = 0·23, *d* = −0·22; *m*_*AUT/TAU*_ = −0·93, 95%-CI: [−2·39,0·53], *p* = 0·21, *d* = −0·25). The three groups aligned more closely at three months (*m*_*IND/TAU*_ = −0·29, 95%-CI: [−2·25,1·66], *p* = 0·77, *d* = −0·06; *m*_*AUT/TAU*_ = 0·26, 95%-CI: [−1·67,2·19], *p* = 0·79, *d* = 0·05).

### Acceptance and harms of the online programme

The acceptance of the online programme was assessed using a 6-point Likert scale (strongly disagree: 0, strongly agree: 5). In the IND and AUT conditions, 61% (*n* = 284 of 468) and 50% (*n* = 188 of 373) of caregivers agreed or strongly agreed that the online programme was helpful, compared with 10% (*n* = 25 of 251) in the TAU condition. In the IND condition, 38% (*n* = 177 of 468) of the caregivers found the messages to be “strongly helpful”, whereas in the AUT condition, only 7% (*n* = 25 of 373) did so.

A total of 11 serious adverse events (IND: *n* = 6; AUT: *n* = 3; TAU: *n* = 2) were reported, exclusively related to severe physical illnesses unrelated to this study, at the four-week and three-month assessments after randomisation. Additional information on serious adverse events and usage data is described in the supplement.

## Discussion

This is to date the largest randomised controlled trial targeting the mental health of informal caregivers of people with depression, and the first to directly compare automated versus individualised psychological support within this setting. It has to be considered that the data collection occurred during the COVID-19 pandemic which may have influenced caregivers’ experiences and potentially amplified their burden. Our results demonstrate that online self-help with either automated or individualised messages statistically significantly reduces the psychosocial distress in caregivers of depressed individuals compared to receiving information material at four weeks after randomisation and over the subsequent two months of follow-up. Caregivers in both active interventions also reported considerable improvement in psychological symptoms, and in their family interaction behaviour in form of reduced criticism and less over-involvement.

This highlights the potential of digital interventions to alleviate the psychological distress of caregiving persons and thereby may reduce their own high risk of mental illness.^6^, ^7^, ^8^ The general efficacy of online programmes for improving mental health is supported by meta-analyses.^27^ Against our expectations, individualised support did not substantially outperform the automated messages. An explanation for this might be that for healthy individuals support with custom-tailored automated messages in an elaborate online programme may have been already sufficient as guidance. Alternatively, it may have yielded even better outcomes to intensify the human guidance by additional telephone or chat sessions.

Despite no statistical differences between the two intervention groups, considerably more IND than AUT users experienced their form of support messages as “strongly helpful”. Interestingly, while psychoeducational support is recommended for significant others in national and international clinical practice guidelines for depression (e.g., NICE,^12^ RANZCP,^12^ NVL^13^), the vast majority of the caregivers in the TAU group evaluated their arm (information leaflet) as not helpful.

Given that 84% of caregivers do not receive information material in German routine care,^28^ the practice guidelines for depression could be supplemented by the recommendation of self-help online programmes such as ours. The German Innovation Fund for example officially recommends the dissemination of our online programme into German healthcare services as a “low-threshold and flexible support service” to national mental health organisations and associations. In the future, the study design could be expanded further to include a stepped-care monitoring approach, in which caregivers who develop mental health problems despite the use of the online self-help programme are offered further care.

In the group of depressed significant others of the caregivers, there was a decrease in symptoms over the measurement period, although no significant group differences between conditions were found. However, the low participation rate among the depressed individuals and the multitude of other possible external factors such as initiation of medication treatment limits the conclusion regarding downstream patient benefits.^29^ Nevertheless, the reduction of psychosocial stress of the caregivers, and the associated improvement in interaction behaviour in the family may be long-term factors for a more favourable disease course of the patient.^9^, ^10^, ^11^ These findings are also in line with a recent meta analysis^30^ on collaborative care emphasising the critical role of significant others involvement, a previously understudied component. Family engagement in collaborative care programmes for depression can range from psychoeducation to active participation in patient care, with clinical benefits for patients' mental health outcomes. In this context, online programmes for caregivers seem particularly suitable, because they offer an easily accessible and flexible to use option.

Regarding strengths of the trial, the study is robust in terms of sample size with *n* = 1640 caregivers from various age groups and genders, which increases the generalisability of the findings. Retention rates were satisfactory throughout the study compared to other online programmes. Comparing the two interventions IND and AUT with a control group allows for an in-depth examination of the specific benefits of each form of support. The results were sustainable three months after randomisation. However, a longer-term follow-up period would be desirable.

Regarding limitations, inclusion of only German-speaking caregivers limits generalisability. Second, recruitment primarily occurred through online channels, which may have led to an overrepresentation of caregivers who are prone to the use of internet tools. Third, the study relied on self-reported outcomes (and was therefore not blinded), which may introduce bias - though this is generally difficult to avoid in digital mental health research. Fourth, the power analysis and resulting sample calculation were not primarily aimed at comparing the support conditions but rather to evaluate those conditions against TAU. Fifth, although supported by sensitivity analyses, our multiple imputation approach may not have fully mitigated the potential biases introduced by the dropout rates. Due to the online format, reasons for dropout could not be recorded, and the nature and extent of this bias remain undetermined. Finally, the intervention effects are rather small and therefore might be considered irrelevant. However, there are several important aspects to adequately contextualise the results of this trial: The effects were observed in a sample of caregivers without current mental disorders – an inclusion criterion that typically yields smaller effects compared to clinical populations. Furthermore, even modest improvements in distress and burden can translate into meaningful enhancements in caregivers' quality of life and resilience. Last, even interventions with small effects can be clinically relevant when scaled up, especially in settings at population level to prevent the onset or deterioration of mental health conditions.

In conclusion, the results of this study demonstrate the efficacy of an interactive online self-help programme in supporting caregivers of individuals with depression. Our outcomes suggest that automated support represents a useful alternative to individualised support, particularly when resources are limited. However, it remains an open research question if under certain circumstances individualised support could be superior to automated support. The findings indicate that targeted interventions for informal caregivers of people with depression not only reduce their psychological distress more effectively than information material alone, but also improve their interaction behaviour with the depressed person. Consequently, these interventions may positively influence both caregivers' and patients' well-being. Besides a planned cost-effectiveness analysis, future studies should evaluate extended long-term effects and the integration of these interventions with potential extensions into routine care systems.

## Contributors

ES and NZ contributed equally as co-first authors. ES, NZ and EG had full access to all study data, verified the underlying data, and are responsible for the decision to submit the manuscript. ES, NZ, ME and EG were the main contributors for the selection and interpretation of key data and drafting this manuscript. ES, CB, CR, KD and EG led the study design, with support from NZ. EG and MG provided expertise on statistical planning, and EG und JS were responsible for the statistical analyses. UH, AE, AM and MS were the main contributors in planning and executing the recruitment strategy. ES, NZ and CB were responsible for study implementation, as well as training and supervision of the support psychologists. All authors provided feedback on the initial draft of the manuscript, read, and approved the final version.

## Data sharing Statement

After de-identification, individual participant data that underlie the results reported in this Article will be shared with researchers who provide a methodologically sound proposal. Proposals should be directed to elisabeth.schramm@uniklinik-freiburg.de; to obtain access to the data, requesters will need to sign a data access agreement.

## Declaration of interests

ES, UH, AM, and EG reported that financing for this trial was provided by a grant from the German Innovation Fund of the Federal Joint Committee (“G-BA Innovationsfonds”, 01VSF19054). ES reported receiving grants and funding from the German Research Council, the Federal Ministry of Education and Research, the Foundation for Mental Health, and the AOK Federal Association during the conduct of the study. EG reported consultancy honoraria from Roche Pharma AG, Germany, and from the Swiss National Science Foundation. KD reported consulting fees from Lundbeck Foundation (Neurotorium) and speaker's honoraria from Janssen-Cilag GmbH. She is a board member of the German Society of Psychiatry (DGPPN) and Freiburg Training Institute for Behavioral Therapy (FAVT GmbH). UH is Chair of the Board of the European Alliance Against Depression (EAAD). No other disclosures were reported.

## Appendix A Supplementary data

Supplementary Material

Trial protocol
