# Supplementary material for: Efficacy of an online self-help programme with automated or individualised psychological support versus treatment as usual for caregivers of people with depression: a randomised, controlled, open-label, superiority trial
Source: Lancet Reg Health Eur. 2025 Dec 11;62:101560. doi: 10.1016/j.lanepe.2025.101560 (PMC12757463; doi:10.1016/j.lanepe.2025.101560)
Supplement: Supplementary Material [file mmc1.pdf]

SUPPLEMENTARY MATERIAL TO

An interactive online programme with automated or individualised psychological support versus treatment-as-usual for caregivers of depressed individuals: A randomised controlled trial

Prof Elisabeth Schramm[1]*, PhD; Nadine Zehender[1], MSc; Christoph Breuninger[1,2], PhD; Prof Ulrich Hegerl[3,4], MD; Anne Elsner[4], MSc; Prof Andy Maun[5], MD, PhD; Marina Schmölz[5], MSc; Christiane Roick, MD[6]; Jörg Sahlmann[7], MD, MSc; Marlon Grodd[7], MSc; Prof Katharina Domschke[1], MA, MD, PhD; Moritz Elsaesser[1], MSc; Erika Graf[7], PhD

[1]Department of Psychiatry and Psychotherapy, Medical Center – University of Freiburg, Faculty of Medicine, University of Freiburg, Freiburg, Germany.
[2]Department of Psychology, Laboratory for Biological Psychology, Clinical Psychology and Psychotherapy, University of Freiburg, Freiburg, Germany
[3]Department of Psychiatry, Psychosomatic Medicine and Psychotherapy, University Hospital Frankfurt, Goethe University Frankfurt (Distinguished Professorship funded by Dr. Senckenbergische Stiftung), Frankfurt am Main, Germany.
[4]German Depression Foundation, Leipzig, Germany.
[5]Institute of General Practice / Family Medicine, Faculty of Medicine and Medical Center – University of Freiburg, Freiburg, Germany.
[6]AOK Federal Association, Berlin, Germany.
[7]Institute of Medical Biometry and Statistics, Medical Center - University of Freiburg, Faculty of Medicine, University of Freiburg, Freiburg, Germany.

*Correspondence to:
Prof Dr Elisabeth Schramm
Department of Psychiatry and Psychotherapy,
Medical Center – University of Freiburg,
Faculty of Medicine, University of Freiburg,
Hauptstr. 5, 79104 Freiburg, Germany
elisabeth.schramm@uniklinik-freiburg.de
Phone: +49 761 270-69670

TABLE OF CONTENT

1. Adverse events	p. 3
2. Recruitment sources	p. 4
3. Sample size derivation	p. 5
4. Statistical analyses	p. 6
5. Results: Comparison IND versus AUT	p. 8
6. References	p. 8
7. Typical sequence of support messages in the AUT and IND condition	p. 10
8. Study protocol	
9. Statistical analysis plan	

1. Adverse events

Adverse events were assessed using the following items:

1. Have there been any serious or distressing changes in your personal life in the past four weeks (e.g., separation, …)?
   If applicable:
   a) What were these changes?
   b) Do you believe these changes are related to your participation in the study?
2. Have there been any serious or distressing changes in your professional life in the past four weeks (e.g., job loss, …)?
   If applicable:
   a) What were these changes?
   b) Do you believe these changes are related to your participation in the study?
3. Have you experienced any new or worsening serious physical symptoms or illnesses in the past four weeks?
   If applicable:
   a) What were these physical symptoms or illnesses?
   b) Do you believe these physical symptoms or illnesses are related to your participation in the study?
4. Have you experienced any new or worsening serious psychological symptoms or mental health disorders in the past four weeks?
   If applicable:
   a) What were these psychological symptoms or disorders?
   b) Do you believe these psychological symptoms or disorders are related to your participation in the study?
5. Have there been any serious illnesses or deaths in your family or close social circle in the past four weeks?
   If applicable:
   a) What were these illnesses or deaths?
   b) Do you believe these illnesses or deaths are related to your participation in the study?
6. Have suicidal thoughts appeared or worsened in your depressed relative in the past four weeks?
   If applicable:
   a) Was there a suicide attempt?
   b) Did your relative survive the suicide attempt?

c) Do you believe these suicidal thoughts or the suicide attempt are related to your participation in the study?

7. Has the depressive disorder of your depressed relative significantly worsened in the past four weeks?
   If applicable:
   a) What has worsened?
   b) Do you believe this worsening is related to your participation in the study?
8. Has your contact with the depressed relative changed significantly in the past four weeks (e.g., change in living situation, substantially more/less contact, …)?
   If applicable:
   a) What has changed?
   b) Do you believe this change is related to your participation in the study?

All caregivers' reports of adverse events were individually reviewed and assessed by designated study staff members in consultation with the principal investigator, according to the GCP (Good Clinical Practice) criteria for serious adverse events. Eleven adverse events were classified as "serious“, including surgery for an unspecified condition, sarcoidosis, severe exacerbation of multiple sclerosis, COVID-19 infection requiring hospitalisation, ulcerative colitis, skin cancer, unspecified cancer, atrial fibrillation requiring hospitalisation, severe pneumonia, multiple sclerosis, and major surgery (unspecified). Of the 11 serious adverse events, 6 occurred in the IND condition, 3 in the AUT condition, and 2 in the TAU condition. All serious adverse events were evaluated - both subjectively (based on reports from the caregivers) and objectively (based on assessments by multiple study staff) - as not related to or caused by participation in the study.

2. Recruitment sources

Data on recruitment sources are only available from January 19, 2022 onward provided by a subset of the sample (904 of 2400 participants). Recruitment sources were assessed using a multiple-response item. The responses are summarised in the following table:

Recruitment Source	Frequencies	Percent
German Depression Foundation	391	43%
Other*	263	29%
Friends or Family	131	14%
Alliance Against Depression	51	6%
General Practitioner	42	5%

Inpatient Stay / Hospital	36	4%
Health Insurance Providers	18	2%
Outpatient Psychotherapist	17	2%
Outpatient Psychiatrist	14	2%
No Response	19	2%

*In the subsequent open-text specification for the response category "Other," 166 participants (18%) reported having become aware of the study through Google, internet searches, or similar online research.

3. Sample size derivation

The target numbers of caregivers to be randomised to the respective intervention and control groups IND:AUT:TAU were changed in a protocol amendment from originally n=500:500:250 (approved by the ethics committee on February 11, 2021) to n=656:656:328 (approved on August 11, 2022). This was done to account for a higher-than-expected rate of missing K-10 outcome measurements at post-intervention, originally anticipated near 30%, which was changed to 39% after the first phase of the trial, both times with the intention to target an expected n=400:400:200 of non-missing primary outcomes, based on the following considerations on statistical power.

A confirmatory comparison of the treatment arms was planned using a pre-specified sequential closed testing procedure. In this approach, hypothesis testing would proceed in a predefined order and would be discontinued after the first non-significant result, in order to control the multiple significance level at 5%. Pairwise comparisons between the randomised groups were to be conducted by calculating two-sided 95% confidence intervals for the difference in mean K-10 changes from baseline. A confidence interval excluding zero would be considered equivalent to rejecting the null hypothesis of equal means, based on an unpaired two-sided t-test at a significance level of 5%. For caregivers of depressed individuals, the expected standard deviation of K-10 scores was assumed to lie in between that of patients (SD: 4·6 points) and students (SD: 7·8 points) seen in the German norm sample.[1] Therefore, a standard deviation of 6·2 score points was expected. Assuming a correlation of 0·5 between baseline and post-intervention K-10 scores, the change from baseline also had an expected standard deviation of 6·2 points. Hence, the effect size in terms of Cohen's d = 0·5 / 0·3 / 0·2 / 0·1 was expected to correspond to a difference between group means of 3·10 / 1·86 / 1·24 / 0·62 K-10 score points.

To demonstrate superiority of interventions, the first and second statistical tests were to reject equalities IND=TAU and AUT=TAU of mean pre-to-post-(T2-T0) K-10 changes at significance level α=5%. They had a power of 1-β≈99% given a medium effect size (Cohen's d) of d=0·5, and of 1-β=93% if d=0·3. Such effect sizes appeared to be realistic based on a single-arm pre-post comparison following a psychoeducation program which yielded d=1·1 for a shorter

version of the primary outcome scale, the K-6 scale.[2] For the third comparison between intervention groups IND and AUT, both superiority of IND over AUT and, to a lesser degree, equality of IND and AUT appeared realistic. The pairwise test to demonstrate superiority by rejecting equality IND=AUT had a power of 1-β=81%, given a small effect size of *d*=0·2. Confirmatory testing was to stop after this comparison. Given equal means IND=AUT (*d*=0), it would also be of interest to reject inferiority of AUT to demonstrate non-inferiority of the less costly intervention AUT compared to the more costly intervention IND at one-sided significance level α=2·5%. Therefore, if the lower limit of the two-sided 95%-confidence interval for the difference of mean K-10 changes from baseline (IND minus AUT) would be greater than -0·62 points (derived as *d*=-0·1), this would be interpreted as non-inferiority. The power to achieve this secondary objective, i.e. to reject such a small inferiority, was 1-β=29%. Higher recruitment targets to achieve greater power were dismissed for reasons of feasibility. The scenarios considered are summarised in terms of standardised group means shifted towards 0 for the TAU group and in terms of expected differences in K-10 means from TAU in the table below (nQuery V8.3.1.0, module MGT0U). Analysis in a linear regression model adjusting for randomisation factors was planned to further increase the statistical power.

Standardised mean IND,AUT,TAU difference in K-10 points	Power to reject a single null hypothesis (n=400:400:200):			
	IND=TAU	AUT=TAU	IND=AUT	AUT inf IND
0·5, 0·3, 0<br>*3·10, 1·86, 0*	99%	93%	81%	<1%
0·5, 0·5, 0<br>3·10, 3·10, 0	99%	99%	5%	29%
0·3, 0·3, 0<br>1·86, 1·86, 0	93%	93%	5%	29%

Notes: inf refers to the null hypothesis that AUT is inferior to IND (*d* -0·1, difference in K-10 points between IND and AUT: -0·62).

4. Statistical analyses

In the primary confirmatory intention-to-treat analysis, all randomised caregivers were analysed in the assigned treatment arms, irrespective of adherence to interventions or discontinuation. The effects of allocation to IND, AUT and TAU with respect to the change in K-10 score from baseline to four weeks after randomisation were estimated and tested in a linear regression model. The model included randomised treatment, K-10 baseline scores, and the remaining randomisation stratification factors as independent variables. Since very few

caregivers of diverse gender were expected, they were assigned alternatingly to the groups of females and males in the primary analysis of intervention effects. Confirmatory comparisons of randomised treatment arms followed a sequential closed testing procedure to ensure a multiple type I error rate of 5%, with descriptive reporting of all subsequent analyses. Tests of equality of means of two treatment arms were based on the two-sided 95%-confidence interval for the difference in mean change from baseline estimated from the linear regression model in the following prespecified order: IND versus TAU, AUT versus TAU, IND versus AUT.

Missing values were replaced by multiple imputation using baseline data and post-baseline information according to a treatment policy strategy. The information explored for imputation included baseline internet literacy scores, post-baseline outcomes and, for IND and AUT, markers for usage of the online support (see 9. Statistical Analysis Plan for details). The results were combined using Rubin's rules for multiple imputation.[3] Secondary outcomes for K-10 at T1 and T3 were also analysed in this way. Sensitivity analyses included a conservative approach where missing outcomes in the IND and AUT arms at T2 were imputed from the TAU group and assessed the impact of adherence.

Although it was hypothesised that IND would be superior to AUT, equality of the less costly intervention AUT compared to the more costly intervention IND was also deemed possible. Therefore, a secondary descriptive analysis tested for non-inferiority of AUT compared to IND. A lower limit of the two-sided 95%-confidence interval for the difference of mean K-10 changes from baseline (IND minus AUT) above -0·62 points (Cohen's d > -0·1) was pre-specified as descriptive evidence for non-inferiority.

Secondary outcomes for K-10 at T1 and T3, and for the other scales at T2 and T3, were analysed without imputation in mixed models for repeated measures, with a compound symmetry covariance matrix to account for correlations between (T1,) T2 and T3 outcomes of the same caregiver or depressed person. The term "longitudinal analysis of covariance, ANCOVA" was also used for these models in some places of the Statistical Analysis Plan (see 9), since modern statistical software actually implements ANCOVA models on the basis of linear mixed models. As in the main primary analysis, independent variables included baseline scores and randomisation stratification factors, plus time-dependent effects of treatment. Further outcome scales and further details of the analysis strategy are described in the full study protocol and the Statistical Analysis Plan (see 9).

Throughout and in line with the CONSORT statement[4], emphasis was planned to be given to the reporting of effect estimators with two-sided 95%-confidence intervals rather than p-values where possible. All analyses presented were planned and performed as prespecified, with estimators for the effect of interventions on the original scale of the outcome scores. In addition, it was decided post-hoc to add effect sizes (Cohen's d) for description. For each outcome scale, these were calculated as the adjusted regression-based between-group difference of

the mean changes from baseline per time point, divided by the pooled empirical standard deviation of observed outcomes, weighted by group sizes at the respective time point.

A *post-hoc* Welch two-sample t-test compared the time spent on programme use among caregivers of the active intervention arms who engaged with the programme (i.e., who used it >0 minutes). Cronbach's alpha was calculated *post-hoc* for baseline K-10, SCL-K-9 and FQ questionnaires in all randomised caregivers, and for baseline PHQ-9 scores of participating depressed persons.

Results from other secondary variables assessed within the study that are not reported here will be published elsewhere. A planned comparison of the results seen in the Facing Depression Together trial with historical controls of face-to-face psychoeducation groups for caregivers of depressive in-caregivers from the multicenter SCHILD study[5] had to be cancelled due to unavailability of the data.

5. Results: Comparison IND versus AUT

As described above, differences between IND and AUT mean K-10 changes from baseline > -0·62 score points were defined as non-inferiority of AUT compared to IND, while differences ≤ -0·62 would be viewed as relevant inferiority. Differences ≤ 0 would imply superiority of the individualised (IND) over the automated (AUT) approach, which would be considered negligible if >-0·62. The comparison between IND and AUT yielded a mean difference of -0·57 K-10 score points (95%-CI, [-1·22, 0·08], *p* =0·0914; final confirmatory comparison). Hence, the point estimate of -0·57 score points suggests a negligible benefit of IND over AUT since, although negative, it falls within range of non-inferiority of AUT. However, neither superiority of IND in the confirmatory approach nor non-inferiority of AUT in the exploratory approach could be demonstrated statistically. This is evident from the 95%-confidence interval, which includes values covering inferiority, non-inferiority, and superiority of AUT compared to IND. Notably, the sample size and power calculations were not primarily aimed at comparing the support conditions but rather to evaluate those conditions against TAU.

6. References

1. Giesinger J, Rumpold MG, Schüßler G. Die K10-Screening-Skala für unspezifischen psychischen Distress. *Psychosomatik und Konsiliarpsychiatrie* 2008; 2(2): 104-11.
2. Katsuki F, Takeuchi H, Konishi M, et al. Pre-post changes in psychosocial functioning among relatives of patients with depressive disorders after Brief Multifamily Psychoeducation: a pilot study. *BMC Psychiatry* 2011;11: 56.
3. Rubin DB. Multiple imputation for nonresponse in surveys. Hoboken (NJ): John Wiley & Sons; 1987.

4. Schulz K, Altman DG, Moher D. CONSORT 2010 Statement: updated guidelines for reporting parallel group randomised trials. *BMC Med*. 2010; 8: 18.
5. Frank F, Wilk J, Kriston L, et al. Effectiveness of a brief psychoeducational group intervention for relatives on the course of disease in patients after inpatient depression treatment compared with treatment as usual – study protocol of a multisite randomised controlled trial. *BMC Psychiatry* 2015; 15(1): 1-11.

7. Typical sequence of support messages in the AUT and IND condition

Typical sequence of support messages in the IND condition:

Hello Lara,
A warm welcome to *Together Through Depression*!
I am your support psychologist from the University Medical Centre Freiburg and will be accompanying you over the next four weeks as you work through the online programme.

From our experience, the programme is most helpful when its content is tailored to the individual, and when family members have the opportunity to clarify any questions or challenges they encounter in applying it.

That is exactly what our message support is for. I am here for you at any time if, for example, you would like to explore one of the exercises from the programme in more depth or adapt it to your situation.

You can also get in touch whenever you want more information on a topic, have questions, or simply wish to share something from everyday life with your family member.

I'm always pleased to hear from you.

Your Support Psychologist, Mrs A.

Hello Lara,
How are you?
I see you've already started working through the online programme - that's great! Have you come across anything helpful yet in the module *Depressive Symptoms in Everyday Life*?

How are things with your partner at the moment?

Your Support Psychologist, Mrs A.

Hello Mrs A.,
Thank you for asking! The part that struck me most was the section describing when it is helpful to motivate and when it's better to take a step back.

My husband has been struggling with recurrent depressive episodes for almost three years now, and sometimes I feel I need to give him a push just to get anything moving. At the same time, I've always been careful not to put pressure on him, and I want to keep it that way.

I find situations difficult where I already suspect in advance that he won't manage something - for example, arranging a repair appointment for the washing machine, which requires time and coordination. On the one hand, I want to leave him his autonomy; on the other, he feels bad when it doesn't work out…. even though the consequences are usually manageable.

Do you have any thoughts on that?

Best regards,
Lara

Hello Lara,
I'm pleased to hear you've worked through *Depressive Symptoms in Everyday Life* so thoroughly - many people don't manage that in such a short time.

I think it's a good sign that you and your husband share responsibilities and that he continues to take on tasks. The question of when to step in with support is an important one - and not always easy to answer.

I would suggest discussing together how you want to handle such moments. The chapter how significant others can avoid *Over-Involvement* (link) explains that taking over tasks can be well-intentioned but may unintentionally lead to greater dependency. You are already approaching this very thoughtfully, but perhaps you'll find further ideas for a conversation there.

The chapter *Solving Problems Together* (link) also offers helpful suggestions for making decisions jointly.

I look forward to hearing how the two of you work this out.

Your Support Psychologist, Mrs A.

Hello Mrs A.,
Thank you for the suggestion, I'll have a look at those chapters.

Best regards,
Lara

Hello Lara,
How are you? Did you have a good weekend?

Perhaps the Eisenhower Principle might also be useful for you: tasks are categorised as *urgent/not urgent* and *important/not important*. You and your husband could discuss which things fall into which category. For *urgent and important*, you could have a sort of contingency plan in place where you step in if needed.

It's important that he generally continues to manage tasks independently so he doesn't slip more into a passive role. You'll also find relevant tips on this in the chapter *Avoiding Over-Involvement* (link).

Wishing you a good start to the week.

Your Support Psychologist, Mrs A.

Hello Mrs A.,
That sounds good. I know the Eisenhower Principle from my job, but I hadn't thought of applying it in everyday life with my husband. I think it could help us to see more clearly who takes on what. Thank you!

Best regards,
Lara

Hello Lara,
I’m glad you’re already familiar with the principle - it may work well in this context too.

How are you feeling at the moment? I suspect you often hold back in order to be there for your husband. What are you currently doing to recharge your own „batteries“?

Your Support Psychologist, Mrs A.

Hello Lara,
There are now two weeks left in the programme.
Have you set yourself any goals for the remaining time? I’m still happy to support you.

Do you already have plans for the weekend?

Your Support Psychologist, Mrs A.

Hello Mrs A.,
My husband has been quite stable over the past few days, and we’ve done quite a lot - we went to a local fair and cooked together, among other things. Through the programme, I’ve been noticing more and more the progress he’s made and how much better we’ve learned to work together. Of course, not everything is perfect, but a lot has settled.

In the coming weeks, I’d like to focus more on my own self-care. One major stress factor for my husband is some changes at his workplace, which have led him to take sick leave several times. After working through the chapter *Avoiding Over-Involvement*, I now have some ideas on how to motivate and support him with this problem without overwhelming or pushing him to much.

Best regards,
Lara

Dear Lara,
I’m very glad to hear your husband is doing better at the moment and that you’ve enjoyed some time together.

I’m also pleased the online programme has helped you to recognise your husband’s progress and how you both manage things together - keeping sight of the positives is an important point that is often overlooked.

It’s great that you’re taking away such practical strategies from the programme and can apply them to your husband’s work stress situation. It’s especially important to keep that balance between supporting him and letting him manage on his own.

Your Support Psychologist, Mrs A.

Dear Lara,
I see you've now worked through almost all of the programme content - fantastic!

What did you find most helpful?
Were you able to take anything from the *Self-care* module for yourself?

Your Support Psychologist, Mrs A.

Hello Mrs A.,
I especially liked that much of the content was presented through concrete, everyday (video) examples. It made it easy to picture myself in those situations and relate them to my own.

I also found the task of creating a weekly plan helpful in structuring my time more consciously. I now make sure to build in free time for myself, so the week doesn't feel so full and overwhelming.

In the *Self-care* module, I found the encouragement to plan specific activities for myself - like meeting a friend for coffee or going out - particularly useful. That's something I had recently neglected.

Best regards,
Lara

Dear Lara,
It's great that you were able to relate the examples so well to your own situation.

Implementing a weekly plan is an important step in finding a good balance. I'm glad to hear you're planning more positive activities again - that will certainly help you maintain your strength in the long run.

From Monday, we begin the final week of the programme. If you have any questions or ideas, now would be a good time.

Your Support Psychologist, Mrs A.

Dear Lara,
How are you? How was your weekend?

I wish you a good start to the last week of our guided time together.

Your Support Psychologist, Mrs A.

Dear Lara,
How are you? I'm impressed that you have completed the programme 100% - really well done!

Our time together comes to an end on Friday. If you still have any questions, please do get in touch.

Your Support Psychologist, Mrs A.

Hello Mrs A.,
Thank you so much for your support over the past few weeks! I don't have any outstanding questions at the moment and will now focus on putting what I've learned into practice in everyday life.

I'm already noticing small improvements and want to keep going on this path.

Once again, many thanks - and all the best to you!

Best regards,
Lara

Dear Lara,
I'm delighted that you've taken so much from the programme and from our exchanges.

Applying it in everyday life is the next important step - and I'm sure you'll keep a good balance between caring for your husband and looking after yourself.

The guided study period is now over, but you can still access all of the content.

Thank you for your openness and for the lively exchange.

I wish you and your husband all the best for the future.

Your Support Psychologist, Mrs A.

Typical sequence of support messages in the AUT condition:

Hello Michael,
A warm welcome to *Together Through Depression*!

It's great that you are here to learn more about depression and about living together with your relative who has depression.

You can use the following four modules in the online programme flexibly:
● Depression and Everyday Life
● Self-Care
● Strengthening the Relationship
● What Do I Need to Know?

You can work through the modules independently of each other. Simply choose an area that appeals to you at the moment and have a look inside.

Hello Michael,
welcome back!

You have recently been working with the module *Depression and Everyday Life*. Everyday life with a person suffering from depression is often difficult! That's why it's great that you are looking at how to deal well with your relative's symptoms.

Many relatives very much want to help the person with depression, and end up taking over a therapeutic role which is usually overwhelming! In this section (link) you will find suggestions on how to stay in your role as a relative and still be able to help.

Hello Michael,

Great that you have already started the programme *Together Through Depression* in the first week!

In the past week, you have spent more than half an hour working with the programme. Excellent!

You will probably be able to benefit even more from the online programme if you invest a little more time.

That is often difficult, because relatives of people with depression already have many extra burdens to carry.

In the module *Self-Care* you will find tips here (link) on how to create more free space in your daily life.

Hello Michael,
welcome back, today we have 3 short tips for you!

- Sometimes it helps to look at your own entries again. Under your user account, select *My Area* – there you will find all your entries in one place.
- In everyday life, it is often difficult to put new resolutions into practice. Reminders in the calendar or in the form of small sticky notes can help.
- You can also talk to your relative with depression about the programme's content. This often creates a sense of connection.

Hello Michael,
welcome back!

You have continued working with the module *Self-Care* over the past week. Great - that is a very important topic!

Here (link) in the *Strengthening the Relationship* section you will find suggestions on how to focus on the positives in your relationship with your relative who has depression. If you find ways to enjoy time together despite the depression, it will help both of you!

Hello Michael,

In the past week, you have spent more than an hour working with the programme. Well done!

Today marks the halfway point of your *Together Through Depression* programme.

I will write to you regularly to let you know how active you were in the past week, and the online programme shows your progress in percent.

The goal is not necessarily to work through all of the content. You know best which topics are important to you and how much time you have!

As a guideline: most relatives need about 1.5 hours to work through one module.

Hello Michael,
welcome back! Great to have you here!

You have now been working with *Together Through Depression* for some time. Many relatives are under a great deal of strain and wish for quick changes. Especially in interpersonal matters, however, it often takes time before you see the first results.

Also pay attention to small changes that show something is moving, and remain patient!

Hello Michael,

Great to see you here again!

Tomorrow the final week of *Together Through Depression* begins.

Try to make good use of this last week for yourself. There are certainly still some interesting topics for you!

Hello Michael,
welcome back to *Together Through Depression*!

You have now been working with the online programme for three weeks. Great that you are still active!

Today you are starting the final week of the programme.

You have surely already worked through some topics and perhaps have already been able to take things away for yourself.

Feel free to have another look around the online programme: is there something you haven’t worked on yet that might still be interesting for you?

Hello Michael,
welcome back!

You have now spent two weeks working on the topic *Strengthening the Relationship*. Very good! We hope you can make use of the suggestions for yourself.

Also take a look at the other modules and topic areas. There you will also find helpful and useful information.

Hello Michael,

You have spent the past four weeks working intensively on the topic of depression as well as living together with your relative who has depression. We very much hope that you have

been able to benefit from the content of the *Together Through Depression* online programme.

After you complete the final questionnaires, the online programme will be freely available to you again. This means you can look at your entries at any time and continue working on important topics.

In two months we will contact you with a final survey to find out whether the online programme has had a lasting effect.

We sincerely thank you for your participation and wish you and your relative all the best!

Your *Together Through Depression* Team

CLINICAL TRIAL PROTOCOL

Facing Depression Together –
Evaluation of an online self-help program with individualized vs automated supportive message system for relatives, significant others, and caregivers of depressed persons

Version 2.1, June 2022

Principal Investigator
Prof. Dr. phil. Elisabeth Schramm
University Medical Center Freiburg

Sponsor
University Medical Center Freiburg

1 **Abbreviations** ... **4**
2 **Administrative information** ... **5**
2.1 Roles and responsibilities ... 5
2.2 Funding ... 5
3 **Introduction** ... **5**
3.1 Background and rationale ... 5
3.2 Objectives and estimand ... 9
3.3 Hypothesis ... 10
3.4 Trial design ... 10
4 **Methods: Participants, interventions and outcomes** ... **10**
4.1 Study setting ... 10
4.2 Eligibility criteria ... 11
4.2.1 Criteria for participation of caregivers ... 11
4.2.2 Criteria for participation of depressed person ... 11
4.3 Interventions ... 11
4.4 Outcomes ... 14
4.5 Participant timeline ... 14
4.6 Sample Size ... 16
4.7 Recruitment ... 17
5 **Methods: Assignment of interventions** ... **18**
5.1 Allocation ... 18
5.1.1 Sequence generation ... 18
5.1.2 Allocation concealment mechanism ... 19
5.1.3 Implementation ... 19
5.2 Blinding (masking) ... 19
6 **Methods: Date collection, management and analysis** ... **19**
6.1 Data collection methods ... 19
6.1.1 General Data ... 19
6.1.2 Psychological variables ... 21
6.1.3 Usage Data of the online program ... 23
6.1.4 Data collection quality ... 23
6.2 Data management ... 25
6.2.1 Storage of data ... 25
6.2.2 Data Transfer ... 26
6.2.3 Data protection/Data security ... 26

6.2.4 Data integrity 28
6.3 Statistical methods 28
6.3.1 Biostatistical planning and analysis 28
6.3.2 Main analysis of primary outcome 29
6.3.3 Sensitivity analysis of primary outcome 30
6.3.4 Supplementary analysis of primary outcome 31
6.3.5 Analysis of secondary outcomes 31
6.3.6 Further supplementary analyses 31
7 Methods: Monitoring 32
7.1 Data Monitoring 32
7.2 Harms 32
7.3 Auditing 33
8 Ethics and dissemination 34
8.1 Research ethics approval 34
8.2 Protocol amendments 35
8.3 Consent or assent 35
8.4 Confidentiality 35
8.5 Declaration of interests 36
8.6 Access to data 36
8.7 Ancillary and post-trial care 36
8.8 Dissemination policy 37
9 References 37

1 Abbreviations

AUT: Online self-help program with automated support

IND: Online self-help program with individual support

TAU: Treatment as usual control condition (conventional treatment under conditions of routine care); in this case written information material for relatives of persons with depression

2 Administrative information

2.1 Roles and responsibilities

- Principal Investigator: Prof. Dr. phil. Elisabeth Schramm, Department of Psychiatry and Psychotherapy, Medical Center–University of Freiburg, Faculty of Medicine, University of Freiburg, Freiburg, Germany
- Study coordination: Dipl.-Psych. Christoph Breuninger & M. Sc. Psych. Nadine Zehender, Department of Psychiatry and Psychotherapy, Medical Center–University of Freiburg, Faculty of Medicine, University of Freiburg, Freiburg, Germany
- Statistical Analysis: Dr. rer. nat. Erika Graf, Institute of Medical Biometry and Statistics, Faculty of Medicine, University Medical Center Freiburg, University of Freiburg, Freiburg, Germany
- Recruitment Support: Prof. Dr. med. Ulrich Hegerl, Leipzig, Vorsitzender der Stiftung Deutsche Depressionshilfe
- Recruitment Support: Prof. Dr. med. Andy Maun & Prof. Dr. med. Wilhelm Niebling, Division of General Practice, Medical Center – University of Freiburg, Faculty of Medicine, University of Freiburg, Freiburg, Germany
- Technical implementation of the E-Mail support and online platform: Prof. Gábor Kovács; Berlin; Prodekan Fachbereich Design, Onlinekonzeption

2.2 Funding

Financing for this trial has been secured by a grant from the “Innovationsfonds zur Förderung von Versorgungsforschung (§ 92a Abs. 2 Satz 1 SGB V)” with the reference 01VSF19054.

3 Introduction

3.1 Background and rationale

Depressive disorders are among the most common mental disorders globally, and are ranked by the WHO as the single largest contributor to global disability (World Health Organization, 2017). In Germany, 4.9 million adults are estimated to suffer from unipolar depression annually (Jacobi et al., 2014). During the course of their lives, a majority of Germans are affected by depression either directly (23%) or indirectly as relatives or close friends (37%, Deutsche Depressionshilfe, 2018). Depressive

disorders are a leading cause of burden of disease (years lived with disability), both globally and especially in developed countries like Germany (Vos et al., 2012). Societal costs are also very high, with an estimated 210,000 work years lost annually in Germany (Robert Koch-Institut, 2018). Direct treatment costs in Germany are estimated at 4.6 billion Euro per year (König, Luppa, & Riedel-Heller, 2010), most of which is attributable to inpatient treatment and medication. Apart from pharmacotherapy, psychotherapeutic treatments are considered first-line treatments for depressive disorders (Meister et al., 2018) as recognized in national guidelines (DGPPN, BÄK, KBV, & AWMF (Hrsg.) für die Leitliniengruppe Unipolare Depression, 2015).

Relatives, significant others and other caregivers of depressed persons are a group mostly neglected by current health care systems, despite the fact that they face increased pressures on in the form of objective and subjective burdens and caring responsibilities for the patients as a result of deinstitutionalization of mental health services (Franz, Meyer, & Gallhofer, 2003). Depression puts a great strain on partnership and family: 84 percent of those affected withdraw from social relationships during a depression, according to a recent representative survey by the German Foundation for Depression (Deutsche Depressionshilfe, 2018). Half of those affected by depression experience problems in their partnership, and in 45 percent of these cases the couple ends up separated. The depressive disorder is associated with considerable subjective and objective stress, even for healthy relatives and partners (Bischkopf, Wittmund, & Angermeyer, 2002; Franz et al., 2003): the vast majority of relatives and partners are concerned about the health (95%) and future (86%) of the depressed person, as well as their own future (66%; Franz et al., 2003). The majority suffers from negative feelings such as guilt, shame or fear of the future and experience physical and psychosomatic complaints. (Frank, Hasenmüller, et al., 2015; Franz et al., 2003). As a result, the relatives' ability to cope with everyday life, their relationships and leisure activities are often impaired. These strains also manifest in the caregivers‘ risk of developing a depressive disorder themselves. The 1-year prevalence of depression is found to be more than doubled among caregivers of depressed individuals in comparison to the general population (Butterworth & Rodgers, 2006; Idstad, Ask, & Tambs, 2010; Wittmund, Wilms, Mory, & Angermeyer, 2002). An international review (Steele, Maruyama, & Galynker, 2010) has shown that relatives of people with bipolar disorder, where depressive episodes usually predominate, have

significantly higher rates of depression and other mental illnesses, combined with an increased health care utilization. Reducing this risk should be a central goal of health policy, because a first depressive episode is followed by a recurrent depressive disorder in at least 50% of cases, and thus leads to further episodes, with the afore-mentioned consequences for those affected and for society. Also, there is evidence suggesting an interaction between the stress experienced by relatives and the course of depression in the patients. A greater burden on relatives significantly increases the risk of recurrences and chronic courses of the disease (Hölzel, Härter, Reese, & Kriston, 2011; Perlick, Rosenheck, Clarkin, Raue, & Sirey, 2001).

Almost one in three relatives say that they are poorly informed about depression (Deutsche Depressionshilfe, 2018). However, lack of knowledge among relatives results in less understanding for the patient, less support and misinterpretation of depressive symptoms such as social withdrawal and reduced emotions. On the other hand, a review (Jacob & Bengel, 2000) concluded that specific information for patients and relatives about the disease depression lead to a better understanding of the disease and a higher treatment satisfaction on the part of the patient. Psychoeducational offers for relatives, which include problem-solving and coping strategies in addition to pure knowledge transfer, can reduce the objective and subjective burden on relatives (Katsuki et al., 2011; Shimazu et al., 2011). After the intervention, the percentage of the relatives showing signs of anxiety and depression could be reduced from 50% to 9%. There is also evidence suggesting that recurrence of depression has been reduced after the relatives' participation in psychoeducational courses (Shimazu et al., 2011): The authors report the recurrence rate at 9-month follow-up to be only 9% in the psychoeducation group compared to 50% in the randomized treatment-as-usual control group. In line with this, a more recent paper reviewing 10 studies on psychoeducation for relatives of depressed individuals showed predominantly positive results both in terms of the relatives' strain and the depressive symptoms of the affected (Brady, Kangas, & McGill, 2017). A multi-center study (SCHILD) on group psychoeducation relatives of persons in inpatient depression treatment has just been completed in Germany, but the results are not published yet (Frank, Wilk, et al., 2015).

The importance of psychoeducation for relatives is also underlined by a recommendation in the current S3 guidelines/NVL "Unipolar Depression" (DGPPN et al., 2015). Despite the importance of such services for relatives, there is a dramatic

undersupply. In a survey of all psychiatric hospitals in Germany, Austria and Switzerland, only about 70% of the hospitals stated that they offered psychoeducation courses for relatives of depressive patients at all (Rummel-Kluge, Kluge, & Kissling, 2015). Moreover, only 13% of the relatives in these clinics took part in these offers. The authors discussed possible reasons for this and mentioned inflexible times of the offers, far travel to reach the clinic, and the fear of stigmatization associated with a visit to the clinic. Frank and colleges (2014) reported similar results from a survey of German acute care hospitals: Only one in three clinics offered groups for relatives of depressive patients, and only one in five relatives participated in these groups (Frank et al., 2014). Hence, only about 5-10% of relatives of depressive patients undergoing inpatient treatment are reached.

In the outpatient setting, the situation of relatives of depressive patients is even worse, as offers for relatives are not included in psychotherapy guidelines. While psychiatric specialist can provide services to relatives that are reimbursed by German public health insurances, this option is hardly used due to organizational obstacles. As only 21% of the first-depressive patients receive an inpatient treatment (Gerste & Roick, 2014), the vast majority of relatives of depressive patients do not have access to psychoeducational services provided by specialists in routine care. Due to this problematic supply situation, self-help-offers are an important additional resource for patients as well as relatives. In this area, digital services have become increasingly important throughout Germany and internationally (Deutsche Depressionshilfe, 2017). For these reasons, an online self-help program for relatives and close friends of persons with depression was developed (www.familiencoach-depression.de). The program was developed based on the current scientific literature by Prof. Schramm's working group at the University of Freiburg Medical Center in cooperation with the German AOK health insurances federal association, with the help of an expert advisory council, and with the involvement of focus groups of depressed individuals and relatives of depressed persons. The program is available freely and free of charge since 9/2018. It is met with great interest and acceptance among users as well as experts (a reference will be included in the new S3 guidelines for unipolar depression). However, the family coach depression is only available as an unaccompanied online program so far.

The efficacy of online-interventions in the prevention of mental disorders is evaluated positively in a recent review of meta-analyses (Ebert et al., 2018), even

though the authors note a comparatively small number of studies. Purely informative online-interventions are found to be significantly less effective than guided interventions (Baumeister et al., 2014). At the same time, it is unclear if therapeutic guidance by highly qualified coaches is more effective than purely practical and motivational guidance (Andersson & Titov, 2014). Attempts to automate human guidance, which would help to economically disseminate preventive interventions to a broad public, are still in their infancy (Kelders, Bohlmeijer, Pots, & van Gemert-Pijnen, 2015). Therefore, in the context of the present study we aimed to develop and evaluate both an individualized message system with support provided by trained psychologists, as well as an automated message system aimed at keeping the users motivated and engaged and thus increase the effectiveness of the measure.

3.2 Objectives and estimand

The objective of the research project is to increase the efficacy of an online self-help program for caregivers[1] of depressed persons by developing and implementing individualized vs automated support via secure e-mail messages. Within this evaluation study the following primary research question will be addressed: Which impact has the four-week usage of the online self-help program with individualized or automated e-mail support vs treatment as usual on the risk of mental diseases in caregivers? In order to achieve this, we strive to determine if a treatment policy assigning caregivers of depressed persons to an individualised or automated online self-help intervention reduces the caregiver’s unspecific mental distress compared to assignment to treatment as usual (TAU), as measured by the change of the K-10 score from baseline to four weeks after randomisation (post-measurement minus baseline). The change in mental distress is considered relevant regardless of whether or not the caregiver adheres to or discontinues the assigned treatment. Treatment arms should be compared with respect to the difference in means of the change in K-10 score.

[1] The self-help program evaluated in this study addresses relatives as well as close friends, colleagues, roommates etc., who give support to a depressed person and are affected by the depressive disorder. In the following, they will be referred to summarily as caregivers.

3.3 Hypothesis

Primary hypotheses: The online self-help program with individualized (IND) or automated (AUT) e-mail support reduces the unspecific mental distress of the caregivers (as an indicator for the risk of mental diseases) as compared to TAU. In a comparison of the two kinds of support, the individualized support will be more effective than the automated support.

Secondary hypotheses and goals:

- The online self-help program with individualized or automated e-mail support reduces the psychosocial burden and the subjective symptom burden of the caregivers as well as the depressive symptoms in the depressed person. It improves depression literacy, interaction behaviour and the well-being of the caregivers.
- Assessing the acceptance and usage of the online self-help program
- Identification of moderators and mediator variables
- Qualitative evaluation of (a) the caregivers' experiences with the online self-help program, the support conditions, everyday life experiences and the interaction with the depressed person as well as potential changes brought about by the intervention, and (b) the depressed persons' experiences of potential changes in the caregivers behavior and their interactions.

3.4 Trial design

The study trial is designed as a randomized, controlled open-label superiority trial with three parallel groups. The randomization will be performed as block randomization with a 2:2:1 allocation (IND:AUT:TAU).

4 Methods: Participants, interventions and outcomes

4.1 Study setting

The study takes place only on the internet platform (www.gemeinsam-durch-die-depression.de). There will be neither additional phone contact nor face-to-face contact. The study is addressed to German-speaking participants in Germany. After registration on the online platform, the online intervention and the messaging system are available in the protected login area of the website.

4.2 Eligibility criteria

4.2.1 Criteria for participation of caregivers

Caregivers can participate in the trial alone or together with their depressed relative, significant other or close friend. If only the caregiver participates in the study, the participant has to provide information on themselves and the depressed person they care for, and both must meet the following in- and exclusion criteria:

Inclusion criteria concerning caregiver:

- age 18 years and older
- sufficient German language skills
- e-mail account and access to the internet
- informed consent
- caring for a depressed person, who
  - is of age 18 years or older
  - has a primary diagnosis of unipolar depressive disorder OR primarily depressive symptoms (according to caregiver)
  - has no primary diagnosis of any other mental disorder (e. g., bipolar disorder, schizophrenia).

Exclusion criteria concerning caregiver:

- suffering from any mental disorder

4.2.2 Criteria for participation of depressed person

Additionally, the depressed person can participate in the study to provide direct information on their symptoms, but only together with the caregiver. In that case, the depressed person has to provide information on and meet the following criteria:

Inclusion criteria concerning depressed person:

- sufficient German language skills
- e-mail account and access to the internet
- separate informed consent

4.3 Interventions

Only the caregiver will receive one of the three interventions. If the depressed person participates as well, he/she only fills in a short questionnaire at each measurement point (and is able to provide additional feedback on the study if desired).

The online self-help program consists of four interactive, independently usable modules (Figure 1). It instructs caregivers on how to deal with common depressive

symptoms (module „Depression und Alltag”). Furthermore, it informs about depression as a mental illness, specific treatment options, critical situations and suicidal tendencies (module „Was muss ich wissen? “) and advises on how to strengthen the relationship between the depressed person and the caregiver (module „Beziehung stärken“). It provides instructions and exercises on how to find the right balance between caring for the depressed person and self-care (module „Selbstfürsorge “). Completing each module will take approximately 1.5 to 2.5 hours per module including homework exercises.

Fig. 1 Structure und modules of the online self-help program

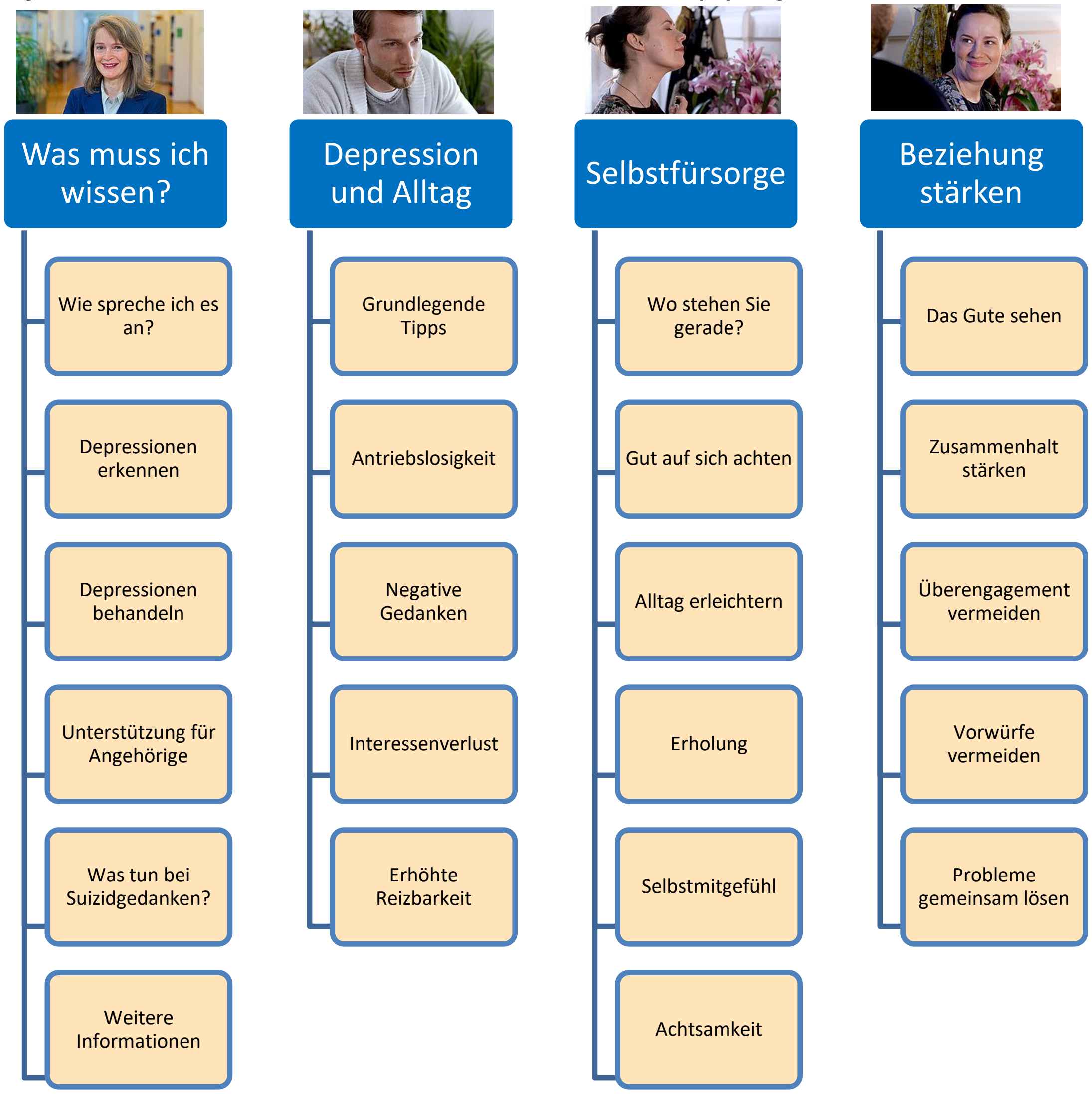

During the study period of four weeks, the online self-help program will be offered in the following three individually randomized variants:

1) *Online self-help program with individual support*: The individual e-mail-support will be implemented by specifically trained psychologists and under regular supervision

with experienced psychotherapists. The psychologist contacts the caregivers three time per week via a secure message system, to give additional information about the modules (e.g. using examples), clarify individual content-related questions and strengthen the motivation of the caregivers in dealing with individual obstacles. For the supporting psychologists, guidelines with instructions and text templates will be developed, to reduce the expenditures of time and costs substantially. Because of its high degree of standardisation, the email-support is much more efficient than other possible formats (e.g. telephone or chat). Since many questions will arise repetitively, text templates will be created gradually, which will reduce personnel costs for the individual support.

2) *Online self-help program with automated support:* The fully automated message system includes regular motivational e-mails, reminders with encouraging messages, and feedback on completed modules. In the development of the automated support system, a focus group consisting of experts in e-mental-health and psychoeducation for relatives was conducted, and caregiver experiences were collected in qualitative interviews in a pilot study.

3) *treatment as usual (TAU) control condition with written information*: In the TAU control condition the caregivers receive a digital version of the patient information leaflet „Depression – Angehörige und Freunde“, which was developed by the ÄZQ based on patient guidelines of unipolar depression.(Ärztliches Zentrum für Qualität in der Medizin (ÄZQ), 2016). On two pages, this document presents concise information on living with the situation, supporting the depressed person, managing crises and caring for oneself. After the study period of three months, the caregivers of the TAU control condition can also use the online self-help program with automated support.

4.4 Outcomes

Primary outcome:

The primary outcome of the study is the unspecific psychological distress as indicator for the risk of mental illness in caregivers (K-10-scale) at post-intervention (T2).

Secondary outcomes:

All outcomes are to be assessed at post-intervention (T2) and at 3-month follow-up (T3)

- The subjective und objective psychosocial burden of caregiving (involvement evaluation questionnaire / IEQ-EU)
- The subjective symptom burden (symptom checklist short version / SCL-K-9)
- The depression literacy (depression literacy test; D-Lit)
- High expressed emotion aspects "critical comments" and "over-involvement" (Familienfragebogen; FFB)
- The well-being (WHO-5) of the caregivers
- Depressive symptoms in the depressed person (patient health questionnaire PHQ-9).

4.5 Participant timeline

Before registration on the website, interested caregivers are provided with written information on the study and an e-mail-address of the study team to answer any remaining questions. Informed consent is obtained from participants by actively selecting a checkbox at the end of the informed consent and data protection summary. Afterwards, caregivers can register on the website by filling in their e-mail-address and responding to a verification e-mail (double opt in). Afterwards, the caregiver will be asked some questions to check the in- and exclusion criteria (Screening). If the caregiver fulfills the criteria, he/she will be included in the study and asked, if the depressed person would like to participate as well. At the caregiver's request, a personalized invitation e-mail is sent to the depressed person, which directs him/her to a study information and informed consent page tailored at depressed participants. Then the caregiver (and if applicable the depressed person) will be asked to complete the pre-intervention questionnaires (T0). After completing his/her questionnaires, the caregiver will be randomized (IND:AUT:TAU; 2:2:1) to one of the three interventions:

1) **Online self-help program with individual support (IND):**

The caregiver can now use the online self-help program for four weeks. He/she receives additional messages from psychologist three times per week, to give additional information about the modules (e.g. using examples), clarify individual content-related questions and strength the motivation of the caregivers in dealing with individual obstacles. The caregiver can contact the psychologist by writing messages in the same secure system.

2) Online self-help program with automated support (AUT):

The caregiver can now use the online self-help program for four weeks. He/she receives additional fully automated messages included regular motivational e-mails, reminders with encouraging messages, and feedback on completed modules. The caregiver cannot reply to the automated messages sent by the online program.

3) treatment as usual control condition with written information (TAU):

The caregiver gets access to the patient information „Depression – Angehörige und Freunde“, which is developed by the ÄZQ based on patient guidelines of unipolar depression. After the study period of three month the caregivers of the TAU-control-condition can also use the online self-help program with automated support.

One week after randomization (T1) the caregiver will be asked to fill in a short questionnaire concerning the psychological distress of the caregivers (K-10). After the intervention period of four weeks, the caregivers and depressed persons fill in the psychological questionnaires again (T2). At this time, there will be additional questionnaire for the caregivers who were randomized to one of the active interventions, asking for the acceptance, adherence to and usage of the online program. All caregivers are also asked, if adverse events occurred during the intervention period. Three months after T0 (T3) the caregivers and depressed persons will fill in the psychological questionnaires again und the caregivers will be asked for occurred adverse events in the last 8 weeks.

At T2 and optionally T3, individual participants (caregivers and depressed persons) will be selected based on stratification criteria and asked whether they would like to take part in a research interview of approximately 60-90 minutes by telephone. The participation in the interview is voluntary and does not affect the general study participation in any way. If the participants are interested in taking part, they will be given an additional study information and consent form for the interview.

The participant’s timeline is shown in the following figure:

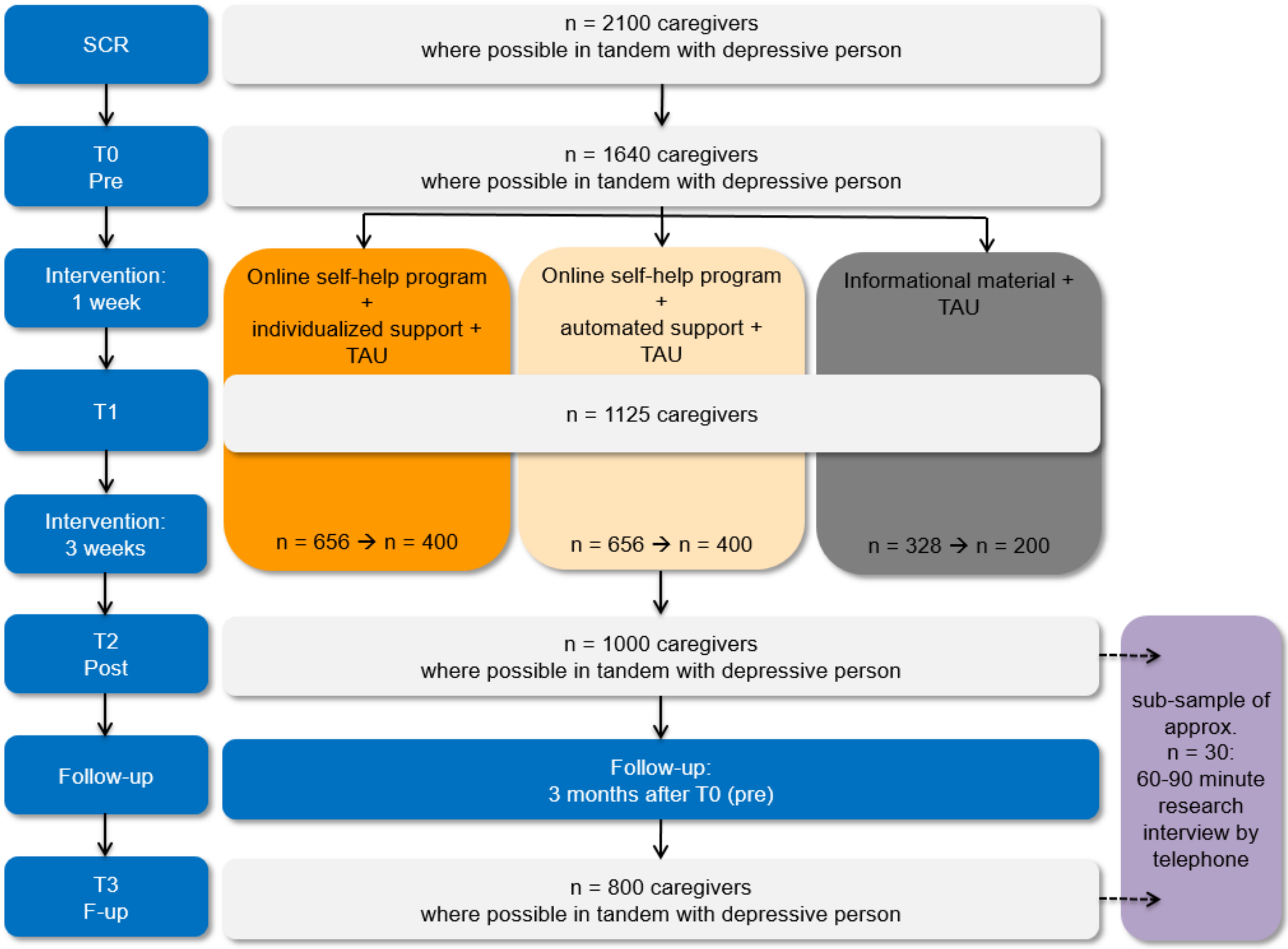

4.6 Sample Size

The numbers of caregivers to be randomized to the respective intervention and control groups IND:AUT:TAU are n=656:656:328. This accounts for an expected 39% rate of missing K-10 outcome measurements at T2 (as estimated from the first phase of the ongoing trial) – yielding an expected n=400:400:200 non-missing primary outcomes – and for the following power considerations. Randomized groups will be compared using a sequential closed test procedure: To guarantee a multiple significance level of 5%, confirmatory statistical testing at nominal significance level α=5% will continue until the first non-significant result. Pairwise comparisons between randomized groups will be performed via calculation of a two-sided 95% confidence interval for the difference of means, for which exclusion of a zero difference is equivalent to rejection of the null hypothesis in an unpaired t-test of equal means at

two-sided significance level α=5%. The first and second statistical test to reject equalities IND=TAU and AUT=TAU of mean pre-to-post-(T2-T0) K-10 changes at significance level α=5% will implement the primary objective by comparing online self-help program interventions with control; they have a power of 1-β≈99% given a medium effect size (Cohen's d) of d=0.5, and of 1-β=93% if d=0.3. Such effect sizes appear to be realistic based on a single-arm pre-post comparison following a psychoeducation program which yielded d=1.1 for a shorter version of the primary outcome scale, the K-6 scale (Katsuki et al., 2011). As regards the third comparison among the two online self-help program intervention groups IND and AUT, both superiority of IND over AUT and, to a lesser degree, equality of IND and AUT can appear realistic; it would be worth-while to reject equality IND=AUT or inferiority of AUT. The pairwise test comparing the two online self-help program intervention groups to reject IND=AUT has a power of 1-β=81%, given a small effect size of d=0.2. Confirmatory testing will stop after this comparison. Given equal means IND=AUT (d=0), it would also be of interest to reject inferiority of AUT (d=-0.1), i.e. to demonstrate non-inferiority of the less costly intervention AUT compared to the more costly intervention IND at one-sided significance level α=2.5%. The power to achieve this secondary objective, i.e. to reject such a small inferiority, is 1-β=29%. Higher recruitment targets to achieve greater power were dismissed for reasons of feasibility. The scenarios considered are summarized in terms of standardized group means shifted towards 0 for the TAU group in the table below (nQuery V8.3.1.0, module MGT0U).

Standardised mean IND,AUT,TAU	Power to reject a single null hypothesis (n=400:400:200):			
	IND=TAU	AUT=TAU	IND=AUT	AUT inf IND
0.5, 0.3, 0	99%	93%	81%	<1%
0.5, 0.5, 0	99%	99%	5%	29%
0.3, 0.3, 0	93%	93%	5%	29%

Notes: inf refers to the null hypothesis that AUT is inferior to IND (d=-0.1).

4.7 Recruitment

Caregivers of depressed persons will be recruited online throughout Germany. As the majority of depressed persons are treated by general practitioners (Gerste & Roick, 2014), general practitioner practices as well as specialist practices for psychiatry and psychotherapy and psychotherapists will be informed and sensitized to the role of caregivers in the treatment of depression. Clinics for psychiatry and psychotherapy will

be included in the recruitment strategy as well, because within in-patient care only the minority of caregivers is able to access psychoeducative offers, so most caregivers are unprepared for the critical phase after the discharge of the depressed patient. We will provide informational material for patients and care-givers (posters, flyers) to interested practices and clinics. Other caregivers will be addressed through online forums, self-help groups for caregivers, self-help contact bureaus, crisis intervention centers and caregivers' associations. Furthermore, information on study participation will be disseminated through 85 regional alliances against depression (Bündnisse gegen Depression) and a patient congress (most recently in Leipzig with more than 1300 participants, half of which caregivers of depressed persons), both organized by the German Depression Assistance Foundation (Stiftung Deutsche Depressionshilfe), as well as through a dedicated informational web page for relatives of depressed persons by the German Depression Assistance Foundation which has several hundred visitors each day. The general public will be addressed through the members' magazines and internet pages of the cooperating health insurance AOK and via advertisements in search engines, social platforms and other media.

The informed consent, delivery of the intervention and data collection will take place on a research platform website set up for these purposes. No information on participants will be processed or communicated during recruitment. Rather, potential participants will be referred to the research website for information and registration.

It is expected that during the twenty-two-month recruiting period about 2100 caregivers will be declared about the study and screened in terms of the inclusion criteria and that 1640 caregivers can be included and randomized within the study.

5 Methods: Assignment of interventions

5.1 Allocation

5.1.1 Sequence generation

For allocation to the intervention and control groups IND, AUT and TAU, a computer-generated random list with a 2:2:1 ratio is used, with stratification based on the participating caregivers characteristics as follows:

- age (years, 18-40, 41-65, ≥ 66)
- gender (female, male, diverse)
- relation with depressed person (parent, child, partner, other)
- K-10-scale (10-22, 23-50).

These characteristics were selected because each variable could plausibly influence participants' response to the interventions. The K-10 cut-off value was chosen based on its ability to differentiate healthy persons from persons at risk of a mental disorder in the German validation study (Giesinger, Rumpold, & Schüßler, 2008). Details of the randomization algorithm will be documented separately and will not be disclosed to the participants.

5.1.2 Allocation concealment mechanism

The allocation sequence is implemented in the designated online platform. The result of randomization is communicated to the caregivers via internet after informed consent, screening, and entry of their baseline data. Randomization lists are only accessible to the technical administrators of the online platform.

5.1.3 Implementation

Randomization lists per stratum are generated by IMBI staff not involved in the trial using R version 4.0.2 or higher and supplied to the technical administrators of the online platform. Participating caregivers enroll themselves into the trial during the online informed consent process and are automatically allocated online to one of the three arms using the caregiver's baseline data for stratification.

5.2 Blinding (masking)

This is an open-label trial, therefore, there is no blinding, neither for participants nor for the study team.

6 Methods: Date collection, management and analysis

6.1 Data collection methods

6.1.1 General Data

By registration on the website the caregiver needs to fill in his/her e-mail-address and user name. Afterwards the caregiver will be asked some questions to check the in- and exclusion criteria (Screening). If the caregiver fulfils the criteria, he/she will be included in the study and asked, if the depressed person would like to participate as well. Then the caregiver will be asked for some general data including sociodemographic data of the caregiver and the depressed person, internet literacy, prior experience with psychoeducation as well as diagnostic and treatment status of the depressed person.

Before the intervention period starts the participant has to fill in the psychological questionnaires at T0 (see 0). If the depressed person participates as well, he/she will be asked to fill in a short questionnaire concerning his/her depressive symptoms. One week after starting the intervention (T1) the caregivers will be asked to fill in a short questionnaire concerning the psychological distress of the caregivers (K-10). After the intervention period of four weeks the caregivers and depressed persons fill in the psychological questionnaires again (T2). At this time there will be an additional questionnaire for caregivers asking for the acceptance, adherence and usage of the online program. All caregivers were also asked, if adverse events were occurring during the intervention period. Three months after T0 (T3) the caregivers and depressed persons will fill in the psychological questionnaires again und the caregivers will be asked for occurred adverse events in the last 8 weeks.

6.1.2 Psychological variables

The psychological variables will be collected by the following questionnaires (or interview), which the caregivers fill in (or participate in) at T0, T1 (only K-10), T2 and T3:

	**Screening**	**T0**	**T1**	**T2**	**T3**
**Caregiver**					
informed consent	x				
Screening – questions concerning in- and exclusion criteria	x				
sociodemographic data and relationship with the depressed person		x			
internet literacy		x			
K-10		x	x	x	x
IEQ-EU		x		x	x
SCL-K-9		x		x	x
D-Lit		x		x	x
FFB		x		x	x
WHO-5		x		x	x
acceptance, adherence and usages questionnaire				x	
adverse events questionnaire				x	x
Qualitative interview				(x)	(x)
**depressed person (if applicable)**					
informed consent	x				
PHQ-9		x		x	x
Qualitative interview				(x)	(x)

K10 Scale: The K10 scale (Kessler et al., 2002; German version: Giesinger, Rumpold, & Schüßler, 2008) assesses non-specific psychological distress during the past 30 days. It includes ten items on a five-point Likert scale and five additional items. The answer options for the first ten items range from one (none of the time) to five (all of the time). Questions begin with “During the last 30 days, about how often did …” and continue with (1) … you feel depressed (2) … you feel tired out for no good reason? (3) … you feel nervous?” The maximum score is 50, indicating high psychological

distress. Ten, the minimum score, indicates the absence of psychological distress. The additional items relate to the frequency of psychological distress in the past 30 days as compared to other times, the performance, the frequency of medical visits and the attribution of physical diseases on the perceived distress.

Giesinger et al. (2008) report good internal consistency of the scale (Cronbachs Alpha ranging from 0.80 – 0.90). To measure convergent validity, satisfactory correlations with related scales could be found (correlation with the State-Anxiety-Scale of the STAI (Laux, L., Glanzmann, P., Schaffner, P., Spielberger, 1981) r = .68 and with the scale GSI of the BSI (Franke, 2000) r = .71)

The Involvement Evaluation Questionnaire: **The Involvement Evaluation Questionnaire**: The IEQ-EU (van Wijngaarden et al., 2000; van Winjgaarden, 1992) assessed the burden on caregivers of mentally ill people in the past four weeks. The present study used the German version of the IEQ-EU (Bernert et al., 2001). The IEQ-EU consists of 31 items, which are assessed on a five-point Likert scale ranging from "never" to "always". Of the 31 items, 27 are grouped into four subscales, namely tension, supervision, worrying, and urging. The internal consistency (Cronbachs Alpha) of the subscales is reported as moderate or good, ranging from 0.71- 0.88, for the German version (Bernert et al., 2001).

Symptom checklist 9-item short version: The psychological burden on caregivers of mentally ill people was assessed by the German version of the SCL-K-9 (Klaghofer, R., Brähler, 2001) a shortened version of the Symptom Checklist 90-R (Derogatis, 1977; Franke, 1992). By using nine items on a five-point Likert scale ranging from 0 (not at all) to 4 (very severe), different psychological symptoms are assessed. Cronbach's Alpha was $\alpha = 0.87$, which stands for a good reliability of the SCL-K-9 (Petrowski, Schmalbach, Kliem, Hinz, & Brähler, 2019).

Depression literacy test: The literacy concerning depression was assessed, using the German version of the Depression Literacy Test (D-Lit) (Freitag et al., 2018; english original: Griffiths, K.M., Christensen, H., Jorm, A.F., Evans, K., Groves, 2004). The D-Lit is a 35-item self-report questionnaire, assessing general information about depressive disorders, for example symptoms, impairments and treatments. Response options are "true", "false" and "don't know". Reliability of the scale is reported as moderate with a Cronbachs Alpha of 0.75 (Freitag et al., 2018).

Family Questionnaire: The level of expressed emotion (EE) was assessed by the German version of the Family Questionnaire (FQ) (Wiedemann, Rayki, Feinstein, &

Hahlweg, 2002) a 20-item self-report questionnaire. On a four-point likert scale, ranging from “never” to “very often” caregivers of patients evaluate the frequency of specific reactions toward the patient. Internal consistency was excellent for the subscale “criticism” (Cronbachs Alpha ranging from 0.90- 0.92 depending on the sample) and good for the subscale “emotional over-involvement” (Cronbachs Alpha ranging from 0.79-0.82 depending on the sample).

WHO-5: The subjective psychological well-being was assessed by the German version of the 5-item World Health Organization Well-Being Index (WHO-5) (WHO, 1998). Response options are coded on a six point scale (0 = never; 5 = all of the time). The internal consistency of the German version of the WHO-5 is excellent (Cronbachs Alpha = 0.92) (Brähler, Mühlan, Albani, & Schmidt, 2007).

If the depressed person participates in the study as well, the person has to reply the following questionnaire at T0, T2 and T3:

Patient health questionnaire: Depressive symptoms were assessed by the German version of the patient health questionnaire (PHQ-9) (Löwe, B., Spitzer, R.L., Zipfel, S., Herzog, 2002; english original: Spitzer, 1999). Subjects indicated for each of the nine items on a four point scale, ranging from “never” to “nearly every day”, whether the symptom has bothered them during the previous 2 weeks.

The PHQ-9 showed excellent criterion validity (medical patients: sensitivity, 95%; specificity, 86%) an good internal consistency (Cronbachs Alpha = 0.88) (Gräfe, Zipfel, Herzog, & Löwe, 2004).

6.1.3 Usage Data of the online program

The following usage data will be collected by the online program for each participant: individual progress in the four modules, dates and frequencies of logins and time spent in the online program. In the condition with individualized support, the correspondence with the psychologist will be stored as well, and after manually removing any potentially identifying information, will be used for content analyses of topics in support messages in pseudonymized form.

6.1.4 Data collection quality

To improve data completeness the participants will get repeated E-Mail reminders, if they don´t fill in the questionnaires at T1, T2 or T3. For completing all measurements, the caregivers get a compensation in the form of a 30€ voucher (10€ for each measurement point, to be traded in at a large number of services).

If participants choose to end participation in the study, they will be asked to provide their reasons. Participants no longer interested in the intervention will nonetheless be asked to complete all measurements.

To promote data quality, data will be monitored during data collection. Data monitoring will consist of blinded reviews of the data for programming errors (e. g., data ranges different than expected) as well as for missing data. If problems are detected, corresponding steps will be taken (e. g., additional efforts to reach participants for each measurement if a large proportion of data is found to be missing).

6.1.5 Collection of qualitative interview data

At T2 and possibly T3, individual participants (caregivers and depressed persons) will be selected based on stratification criteria (based on theoretical sampling with both a priori criteria such as type of relationship to depressed person as well as criteria emerging in the analysis process) and interviewed by telephone for approximately 60-90 minutes. The interviews are conducted by trained psychological personnel using a semi-structured interview guideline. The contents of the interviews with caregivers focus on the experiences with the online self-help program, the support conditions, everyday life experiences and the interaction with the depressed person as well as potential changes brought about by the intervention. The contents of the interviews with depressed persons focus on the depressed persons' experiences of potential changes in the caregivers' behavior and their interactions. The semi-structured interview form is intended to capture the subjective perception of the participants. It is characterised by a predetermined catalogue of questions with the flexibility to elaborate topics that arise during the interview or change the order of questions to follow the flow of the conversation. The interview consists of a warm-up phase and five thematic blocks. After the general experiences with the coach and the supportive messages, specific experiences with the topic areas of all four modules will be asked. There are a few narrative prompts for each thematic block, supplemented by follow-up questions that are only explicitly asked if the respective aspects do not appear in the participants' spontaneous narrative or report. The content of the interviews is recorded in the form of audio recordings. Before further analysis, the audio recordings are transcribed into text, removing or changing names and other potentially identifying information to achieve pseudonymized data.

6.2 Data management

6.2.1 Storage of data

All data collected in the context of this study will be collected electronically only. Descriptive data of the participants and usage data (e.g. login duration and frequency) will be recorded, as well as data from questionnaire surveys. The data collection takes place on the website of the online self-help-program, which is created and operated (hosting, account management) by the consortium partner H6 Kommunikationsagentur (Geusenstraße 8, 10317 Berlin, Germany) under the leadership of Prof. Gabor Kovacs. For technical and administrative processes H6 Kommunikationsagentur has access to all data collected in the study. All data collected in the study will be stored on a server of the company Mittwald CM Service GmbH & Co. KG (Königsberger Straße 4-6, 32339 Espelkamp, Germany).

Compliance with the European General Data Protection Regulation (GDPR: EU Regulation 2016/679) is secured through a data processor agreement (AV-Vertrag) between Mittwald and the University Clinic. The same applies for other potential contractors of the consortium partners, while shared and individual responsibilities between the consortium partners H6 Kommunikationsagentur and the University Clinic will be defined in a contract of shared responsibilities (according to Art. 26 DS-GVO).

Data files will be archived in order to comply with the data protection requirements and deleted completely after 10 years. The required archiving periods of at least five years after publications will be adhered to.

The correspondence between the particpants in the condition with individualized support and the psychologist will be transferred by encrypted connection to secure clinic servers for storage and further processing. Before the evaluation of this data, the correspondence are checked by study staff to see whether information appears that allows conclusions to be drawn about the caregiver or the depressed person (e.g. names, addresses, ...). If this is the case, the information is deleted from the correspondence.

The contents of the interviews are divided into sensitive audio recordings on the one hand, which remain on protected clinic servers, and pseudonymized transcripts (names and other information that make a person identifiable are changed during transcription) on the other hand for the further research process. Strict separation of the subjects' names or audio recordings and the other data collected ensures pseudonymized data processing. In case that subjects request a complete deletion of

their own data, all personal data will be deleted. Accordingly, deletion includes all demographic data, contact data and data of inclusion and exclusion criteria as well as potential interview recordings. Questionnaire data, pseudonymized psychological correspondence and transcribed interview data, that has been produced until then, will be analyzed completely anonymized.

6.2.2 Data Transfer

The stored data will be compiled for analysis by the consortium partner H6 Kommunikationsagentur and transferred only to the Institute of Medical Biometry and Statistics of the University Medical Center Freiburg (IMBI) and the study direction. Data transfer to other parties is not intended.

In order to analyse the data, the data records will be transmitted to the Institute of Medical Biometry and Statistics in pseudonymized form. During data collection the Institute of Medical Biometry and Statistics gets access to the data, to monitor the data quality. For further monitoring, the principal investigator can request a data transfer of the pseudonymized data at any time. Data transfer will be carried out only electronically via encrypted data connections and only on the instructions of the principal investigator.

6.2.3 Data protection/Data security

To participate in the study, the participants register with a confirmed e-mail address and a secure password. The use of the online program, messaging system and the associated data collection will take place exclusively in the protected login area of the online program. All data collected will be stored by the Mittwald company. The Mittwald Company undertakes to perpetually treat the collected data confidentially and to use it only for the purpose of conducting the present study. Personal data will be processed in such a way that the data can no longer be assigned to a specific subject without additional information (pseudonymization).

The company Mittwald gives a written undertaking to comply with the European General Data Protection Regulation (GDPR: EU Regulation 2016/679) and with the technical and organizational measures being necessary and appropriate for their compliance, which are as follows:

No unauthorized access to technical facilities	– The company building is divided into different areas of access.<br>– Visitors must register at the reception and are picked up by their contact person.<br>– Access to all data processing systems will be completely denied to unauthorized persons.<br>– The access of any person (including employees) must be approved in advance by authorized personnel and is verified by checks on persons.<br>– All accesses and premises of the data processing systems will be monitored by cameras and controlled by electronic locking systems.<br>– All access will be recorded.
Access controls to important facilities (hardware, operating systems, software)	– The access to the data processing systems is secured by a user and rights administration. Employees can only view, use, process or delete the data required for their tasks.<br>– Accesses to the data processing systems will be logged.<br>– When leaving the workplace, the system is blocked by a screensaver and can be unlocked only by entering the password.<br>– Accordingly, at the time of recruitment every employee is committed to confidentiality and compliance with data protection. A violation would result in instant dismissal and a criminal complaint.
Control of data transmission	– Personal data will be transmitted only electronically via encrypted data connections, so that they cannot be read, copied, changed, or removed by unauthorized persons.

	– Data media that are no longer required or defective will be disposed of by a certified company.
Data Transfer	All Data transfer will be encrypted (encrypted Internet connections using TLS/SSL).

If, despite the detailed technical and organizational measures taken, there should be a serious disturbance of operating procedures, a suspicion of data protection violations or other irregularities in the processing of personal data, the consortiums partners are obliged to inform the study direction immediately and to jointly decide on further proceedings.

All data processing will be carried out in Germany or a member state of the European Union only.

Mittwald Company also implements a data protection management system for the regular monitoring, assessment and evaluation of data protection and the effectiveness of the established technical and organizational measures.

6.2.4 Data integrity

To ensure data integrity, it can be checked retrospectively at any time whether and by whom personal data has been entered, changed or removed in data processing systems. The entry, modification or deletion of personal data is logged with the identification of the responsible employee.

Additionally, the protection of personal data against accidental destruction or loss is ensured (e.g. by RAID systems, replacement hardware, surge protection, UPS systems, emergency generator, extinguishing systems). Also, measures will be taken to ensure the quick restoration of availability and access to personal data in the case of a physical or technical incident (e.g. by daily backups).

6.3 Statistical methods

6.3.1 Biostatistical planning and analysis

Before the start of the final analysis, the details of statistical analysis left open in the trial protocol will be described in a statistical analysis plan (SAP). At the latest, the SAP will be completed during a blinded review of the trial data, i.e. without looking at the randomized intervention for individual caregivers. If the SAP contains any changes to the analyses outlined in the trial protocol, they will be marked as such, and reasons for amendments will be given.

Throughout, emphasis will be given to reporting of effect estimators with two-sided 95%-confidence intervals rather than p-values whenever possible, which will be considered statistically significant if below 5% for tests of equality. However, confirmatory statistical testing will be restricted to the analysis of the primary outcome, to be followed by descriptive reporting of other trial results.

All programming for statistical analysis will be performed with the Statistical Analysis System (SAS) or with R.

6.3.2 Main analysis of primary outcome

Model: The primary efficacy analysis will be performed in the full analysis set (FAS) according to the intention-to-treat (ITT) principle. Therefore, all randomized caregivers will be analysed in the assigned treatment arms, irrespective of treatment adherence and whether they refuse or discontinue the intervention. The effects of allocation to IND, AUT and TAU with respect to the primary endpoint, change in K-10 score from baseline to four weeks after randomisation, will be estimated and tested in a linear regression model, and the corresponding two-sided 95%-confidence intervals will be reported. The model will include randomized treatment (IND, AUT, TAU), age (years, 18-40, 41-65, ≥ 66), gender (female, male; for diverse, see end of paragraph), caregiver's relation with depressed person (parent, child, partner, other), and K-10 baseline scores as independent variables. Since very few caregivers of diverse gender are expected, they will be assigned alternatingly to the groups of females and males in the primary analysis of intervention effects. They will be analysed separately in secondary analyses exploring the effect of gender.

Sequential testing: Following a sequential closed testing procedure to ensure a multiple type I error rate of 5%, confirmatory comparisons of randomized treatment arms will proceed until the first occurrence of a non-significant result at the nominal 5% level, followed by descriptive reporting of all subsequent analyses. The two-sided tests of equality of means of two treatment arms will be based on the two-sided 95% confidence interval for the difference in mean change from baseline estimated from the linear regression model. Treatment arms will be compared in the following order: IND versus TAU, AUT versus TAU, IND versus AUT. Although it is hypothesized and subjected to confirmatory testing that IND is superior to AUT, equality is also deemed possible. In a secondary descriptive analysis, demonstration of non-inferiority of AUT compared to IND will also be attempted. If the lower limit of the two-sided 95%-confidence interval for the difference of mean K-10 changes from baseline (IND minus

AUT) is greater than -0.62 points, this will be interpreted as non-inferiority. The non-inferiority margin was derived as follows: For caregivers of depressed persons, the expected standard deviation of K-10 scores is in between that of patients (SD: 4.6 points) and students (SD: 7.8 points) seen in the German norm sample (Giesinger et al., 2008). Therefore, a standard deviation of 6.2 score points is expected. Assuming a correlation of 0.5 between baseline and post-intervention K-10 scores, the change from baseline has also a standard deviation of 6.2 points, and using Cohen's d=0.1 as non-inferiority margin gives 0.62 points.

Handling of missing values: Multiple imputation will be employed to replace missing values. Imputation will be done per randomized treatment arm, using baseline data and post-baseline information according to a Treatment Policy Strategy as described by Polverejan and Dragalin (2020) and Guizzaro et al. (2020). The results will be combined using Rubin's rules for multiple imputation. The details will be fixed in the statistical analysis plan.

Assumptions: The resulting estimators assume that caregivers with missing outcome data have outcomes similar to those of subjects with similar baseline data and treatment adherence who provide their outcome data.

Interim analysis: As described in section 7.1, an interim analysis is not considered necessary for this study. The lead investigator will monitor the progress of the study with regard to safety-relevant development as detailed in section 7.2.

6.3.3 Sensitivity analysis of primary outcome

To evaluate the robustness of the main analysis with respect to the assumptions on missingness, an alternative multiple imputation model will be applied for sensitivity analysis. It will impute the outcomes that are missing due to study withdrawal such that outcomes in the IND and AUT arms are assumed to be similar to those observed in the TAU group. The details will be fixed in the statistical analysis plan.

To evaluate the robustness of the comparison of IND versus AUT in the main analysis with respect to adherence, the main analysis will be repeated in subpopulations of adherent caregivers. Non-inferiority will be concluded if the lower limit of the corresponding two-sided 95%-confidence interval for the difference of mean K-10 changes from baseline (IND minus AUT) is greater than -0.62 points. The details will be fixed in the statistical analysis plan.

6.3.4 Supplementary analysis of primary outcome

In order to assess the consistency of the treatment effect in relevant subgroups (EMA-CHMP, 2019), exploratory analyses will be performed using all variables for grouping that were used for stratified randomization (i. e. age, gender, relation with depressed person, pre-intervention (T0) K-10 score) as well as internet literacy.

Further analyses will explore possible moderators (measured at baseline, e. g., possible effects of features of the depressed person such as duration of illness or type of relation with the depressed person) and mediators (e. g. adherence to randomized interventions, importance of changes in interaction behaviour for treatment effects on participants K-10 scores) on primary and secondary outcomes. These analyses will be conducted as part of PhD theses and will be pre-specified elsewhere at a later time.

6.3.5 Analysis of secondary outcomes

Secondary outcomes at T2, four weeks after randomization, and T3, three months after randomization, will be evaluated in a linear mixed model per outcome scale, with a compound symmetry covariance matrix to account for correlation between T2- and T3-outcomes of the same caregiver or depressed person. Independent variables will be those of the main primary analysis, plus time point (T2, T3). The details will be fixed in the statistical analysis plan.

Subgroup analyses, moderator and mediator analyses will be performed as described for the primary outcome above.

Furthermore, the results seen in the Facing Depression Together trial will be compared to historical controls of face-to-face psychoeducation groups for caregivers of depressive in-patients from the multicentre SCHILD study (Frank, Wilk, et al., 2015).

6.3.6 Further supplementary analyses

Demographic and other baseline data will be summarised descriptively by randomized arm in the FAS. Continuous data will be summarised by arithmetic mean, standard deviation, minimum, 25% quantile, median, 75% quantile, maximum, and the number of complete and missing observations. If appropriate, continuous variables can also be presented in categories. Categorical data will be summarised by the total number of patients in each category and the number of missing values. Caregivers frequencies are displayed as valid % (number of patients divided by the number of patients with non-missing values).

All data items needed for reporting of trial results in compliance with the CONSORT (Schulz, Altman, & Moher, 2010) and CONSORT-EHEALTH (Eysenbach & CONSORT-EHEALTH Group, 2011) statements will be evaluated.

6.4 Qualitative methods

Based in the interview transcripts, a qualitative content analysis is carried out. The focus of the qualitative analysis is on the subjective perception of the interviewed person. Depending on the research question, an appropriate method within the grounded theory framework is selected. Our research group is experienced in both constructivist grounded theory analyses (Charmaz, 1996, 2006) and core sentence ("Kernsatz") analyses (Burkhardt, 2016; Leithäuser & Volmerg, 1988). For the messages between participants and psychologist from the individual support condition, an appropriate form of qualitative or quantitative content analysis is chosen for each research question.

7 Methods: Monitoring

7.1 Data Monitoring

Following the guideline on data monitoring committees (European Medicines Agency [EMA]) Committee for Medicinal Products for Human Use [CHMP], 2005), neither a data monitoring committee nor interim analyses are considered necessary for this study, given the non-patient participants, short intervention as well as study time and low risk of harm expected from the intervention. The lead investigator will monitor the progress of the study with regard to the safety-relevant development as detailed in section 7.2.

7.2 Harms

The objective of this online-coach is to effect a positive change of caregivers' psychological burden and everyday life. Each change includes a risk for undesirable side effects. Side effects can be impacts on everyday life, interpersonal relationships (e.g. conflicts due to caregivers setting more boundaries towards the patients) and increased awareness of the burdens. Moreover, a temporary burden through the active analysis of actual stress factors is possible. For risk assessment (serious) adverse events ((S)AE) will be defined ahead of the study and recorded during the course of

studies. (Serious) adverse events are the occurrence or deterioration of psychiatric symptoms of the caregiver or the depressive person.

In case of a (S)AE there is an emergency button on the website of the online program, where the participant can find information and contacts of emergencies services and will be asked to contact them.

If (S)AE occur in the psychological message system contact or if participants contact a study member in case of an (S)AE, the lead investigator will be informed of the (S)AE immediately and will decide on the further procedure (e.g. study exclusion and outpatient or inpatient treatment recommendations).

For the following reasons, the intervention will be ceased in subjects of the study:

- Revocation of the subject's declaration of consent
- If, in the opinion of the lead investigator, further participation in the study would have harmful effects on the subject or the depressive person
- In case of new exclusion criteria
- If the emotional burden for the subject is too serious to cope with it himself, and intensive (professional) support is needed

Exclusion is decided by the lead investigators, in case of fulfilling one of the mentioned criteria. The reason for exclusion will be recorded. If needed, excluded subjects will be supported in searching further consulting or treatment. Also, if the subject revokes the informed consent, the subsequent reason will be recorded.

The lead investigator will monitor the progress of the study with regard to safety-relevant development. The lead investigator will discontinue the study, if:

- the cost-benefit ratio for the caregiver or the depressive ill person changes significantly
- there are indications, that the security of the caregiver or the depressive ill person is no longer guaranteed
- the continuation of the study is no longer justified by ethical or medical reasons
- the questions of the study can be clearly answered by results of another study

7.3 Auditing

Since the trial is performed in non-patient participants (caregivers) in combination with a short intervention as well as study time and low risk of harm expected from the intervention, there will be no external audits.

8 Ethics and dissemination

8.1 Research ethics approval

Through working on the online self-help program and the messaging system the participating caregivers are expected to get better and more confident handling with the diagnosis depression and with the interpersonal interaction with the depressed person. Information and help is given, which applies in everyday life and the relationship. Also, the caregiver´s self-care is strengthened. Concerning the co-participating depressive person, it is expected, that trough the caregiver´s participation everyday interaction is getting more useful and less confrontational. Hence, it results a relief for the depressed person. As the online self-help program is a supportive and informing online-intervention, we expect that participation has a positive impact on everyday life and on the mental burden of the participating caregivers. In certain circumstances it may happen, that some subjects do just less or not benefit from the online-intervention. Since the results of this study help to better understand the burden of caregivers of depressive persons, all subjects help to improve possibilities for supporting caregivers of depressive persons.

The objective of this online self-help program is to effect a positive change of caregiver's psychological burden and everyday life. Each change includes a risk for undesirable side effects. Side effects can be impacts on everyday life, interpersonal relationships (e.g. conflicts due to more delimitation) and experiencing of the burden. Moreover, a temporary burden through the active analysis of actual stress factors is possible. Subjects will be informed about all potential side effects by the informed consent.

During the whole study period, subjects can, if required (e.g. in case of a serious event), call a study member – or out of working hours – the doctor of the university clinic of psychiatry and psychotherapy, Freiburg.

Subjects will be informed about the study and all measures within the study. The participant consent (double opt-in) must be obtained before any trial-specific tests. Participation can be ceased at any time without necessarily giving any reason and without any loss of benefits in case of a (further) treatment at the university clinic Freiburg (independence of study and treatment).

The online self-help program is developed in accordance with the S3-guidlines of unipolar depression, which recommends psychoeducation for caregivers. The study is to be conducted according to all legal requirements, as well as ethic and medical

principals (Declaration of Helsinki, Good Clinical Practice, Data Protection Law). The Declaration of Helsinki is the standard for the medical and psychological ethic of the university clinic Freiburg.

8.2 Protocol amendments

Any modifications to the protocol which may impact on the conduct of the study, potential benefit of the participant or may affect participant safety, including changes of study objectives, study design, patient population, sample sizes, study procedures, or significant administrative aspects will require a formal amendment to the protocol. Such amendment will be approved by the Ethics Committee prior to implementation.

8.3 Consent or assent

After the registration on the website, the participant will be informed that participation is voluntary and that he/she may withdraw at any time without having to give reasons and without penalty or loss of benefits to which the participant is otherwise entitled.

The participant consent must be obtained before any trial-specific tests.

By agreeing the consent form, the participant agrees to voluntarily participate in the trial and declares that he/she agrees to the recording of personal data for the trial after pseudonymization.

Afterwards the participant will get the informed consent via E-Mail.

8.4 Confidentiality

The pertinent provisions on data protection must be fully complied with.

The study participants will be informed of the purpose and extent of the collection and use of personal data.

Findings obtained in the course of the trial will be stored on electronic media and treated in strict confidence. For the protection of these data, technical and organizational measures have been taken to prevent disclosure to unauthorized third parties. For example, the participant data will be captured in pseudonymized form (participant ID) throughout the documentation and evaluation phase. The participant IDs will be generated randomly, such that no information on the participant can be extracted from them (i. e., neither order of enrollment nor name initials etc. will be visible). Access to the identification list (connecting IDs and names etc.) is restricted to

the principal investigator. Where technical staff needs full access to the underlying databases, viewing of the identification list is precluded by organizational measures.

8.5 Declaration of interests

Prof. Schramm declares no potential conflicts of interest.

8.6 Access to data

The investigators grant access to participant files for verification of proper documentation of study data as required by law. The laws on data confidentiality (Art. 6 Abs. 1 lit. A DSGVO, LDSG Baden-Württemberg) apply in full. Persons authorized by the principal investigator are allowed to verify data.

No individual participant data is shared with the funding body or cooperating health insurance. The reporting duties towards the funding body are fulfilled with aggregated data (e. g. means, standard deviations) and statistical analyses.

8.7 Ancillary and post-trial care

As detailed in section 7.2, only mild and transient harms are plausibly expected from the planned interventions. Participants who encounter serious adverse events during the course of the study will be assisted in finding appropriate treatment where necessary.

8.8 Dissemination policy

Before recruitment and data collection starts, the trial will be registered at www.drks.de. Study results will be published in accordance to the criteria of the CONSORT-Statement. At least within one year of termination of the study, a manuscript for publication will be finalized. Any formal presentation or publication of data collected as a direct or indirect result of this trial will be considered as a joint publication by the investigators. It requires the agreement of the coordinating investigator and the co-principle investigator. Authorship will be determined by mutual agreement. The results of the study may be presented during scientific symposia or published in a scientific journal only after review and written approval by the coordinating investigator and the co-principle investigator. Investigators from the participating trial sites agree not to engage in presentations based on data gathered individually or by a subgroup of centers before publication of the first main publication, unless this has been agreed otherwise by all other investigators, the coordinating investigator and the co-principle investigator.

9 References

Andersson, G., & Titov, N. (2014). Advantages and limitations of Internet-based interventions for common mental disorders. *World Psychiatry*, *13*(1), 4–11. https://doi.org/10.1002/wps.20083

Ärztliches Zentrum für Qualität in der Medizin (ÄZQ). (2016). *Patienteninformation: Depression - Angehörige und Freunde [auf Basis der Patientenleitlinie „Unipolare Depression"]*. https://doi.org/10.6101/AZQ/000322

Bernert, S., Kilian, R., Matschinger, H., Mory, C., Roick, C., & Angermeyer, M. C. (2001). Die Erfassung der Belastung der Angehörigen psychisch erkrankter Menschen - Die deutsche Version des Involvement Evaluation Questionnaires (IEQ-EU) -. *Psychiatrische Praxis*, *28*(2), 97–101. https://doi.org/10.1055/s-2001-17792

Bischkopf, J., Wittmund, B., & Angermeyer, M. C. (2002). Alltag mit der Depression des Partners. *Psychotherapeut*, *47*(1), 11–15. https://doi.org/10.1007/s00278-001-0194-5

Brady, P., Kangas, M., & McGill, K. (2017). "Family Matters": A Systematic Review of the Evidence For Family Psychoeducation For Major Depressive Disorder. *Journal of Marital and Family Therapy*, *43*(2), 245–263. https://doi.org/10.1111/jmft.12204

Brähler, E., Mühlan, H., Albani, C., & Schmidt, S. (2007). Teststatistische Prüfung und Normierung der deutschen Versionen des EUROHIS-QOL Lebensqualität-Index und des WHO-5 Wohlbefindens-Index. *Diagnostica*, *53*(2), 83–96. https://doi.org/10.1026/0012-1924.53.2.83

Burkhardt, T. (2016). Identität und Migration. Auf der Suche nach Schutzfaktoren mit Problemzentrierten Interviews (PZI) und der Kernsatzmethode. In *Qualitative Methoden in der Sozialforschung* (pp. 145–151). Berlin, Heidelberg: Springer Berlin Heidelberg. https://doi.org/10.1007/978-3-662-47496-9_16

Butterworth, P., & Rodgers, B. (2006). Concordance in the mental health of spouses: analysis of a large national household panel survey. *Psychological Medicine*, *36*(05), 685. https://doi.org/10.1017/S0033291705006677

Charmaz, K. (1996). The Search for Meanings- Grounded Theory. In J. A. Smith, R. Harré, & L. van Langenhove (Eds.), *Rethinking Methods in Psychology* (pp. 27–49). London: Sage Publications.

Charmaz, K. (2006). *Constructing grounded theory*. Thousand Oaks: SAGE.
Derogatis, L. R. (1977). *SCL-90-R, administration, scoring & procedures manual-I for the R(evised) version*. Baltimore: John Hopkins University School of Medicine.
Deutsche Depressionshilfe. (2017). Deutschland-Barometer Depression. Volkskrankheit Depression – So denkt Deutschland. Retrieved from https://www.deutsche-depressionshilfe.de/presse-und-pr/downloads?file=files/cms/downloads/studienergebnisse_depression_so-denkt-deutschland.pdf
Deutsche Depressionshilfe. (2018). Befragung „Volkskrankheit Depression – So denkt Deutschland“. Retrieved December 18, 2018, from https://www.deutsche-depressionshilfe.de/presse-und-pr/downloads?file=files/cms/downloads/Barometer 2018/barometer-depression_grafikband-zum-download.pdf
DGPPN, BÄK, KBV, & AWMF (Hrsg.) für die Leitliniengruppe Unipolare Depression. (2015). *S3-Leitlinie / Nationale VersorgungsLeitlinie Unipolare Depression – Langfassung, 2. Auflage. Version 5. 2015*. https://doi.org/10.6101/AZQ/000364
Ebert, D. D., Van Daele, T., Nordgreen, T., Karekla, M., Compare, A., Zarbo, C., … Baumeister, H. (2018). Internet- and Mobile-Based Psychological Interventions: Applications, Efficacy, and Potential for Improving Mental Health. *European Psychologist*, *23*(2), 167–187. https://doi.org/10.1027/1016-9040/a000318
European Medicines Agency (EMA) Committee for Medicinal Products for Human Use (CHMP). (2005). Guideline on data monitoring committees (Doc. Ref. EMEA/CHMP/EWP/5872/03 Corr).
European Medicines Agency (EMA) Committee for Medicinal Products for Human Use (CHMP). (2019). Guideline on the investigation of subgroups in confirmatory clinical trials (Doc. Ref. EMA/CHMP/539146/2013).
Eysenbach, G., & CONSORT-EHEALTH Group. (2011). CONSORT-EHEALTH: Improving and standardizing evaluation reports of web-based and mobile health interventions. *Journal of Medical Internet Research*, *13*(4), e126. https://doi.org/10.2196/jmir.1923
Frank, F., Hasenmüller, M., Kaiser, M., Ries, Z., Bitzer, E., & Hölzel, L. (2015). Psychoedukative Gruppen für Angehörige von depressiv Erkrankten: Analyse des Informationsbedarfs durch eine Fokusgruppenuntersuchung. *PPmP - Psychotherapie · Psychosomatik · Medizinische Psychologie*, *65*(11), 426–433. https://doi.org/10.1055/s-0035-1555787
Frank, F., Rummel-Kluge, C., Berger, M., Bitzer, E. M., & Hölzel, L. P. (2014). Provision of group psychoeducation for relatives of persons in inpatient depression treatment - a cross-sectional survey of acute care hospitals in Germany. *BMC Psychiatry*, *14*(1), 1–9. https://doi.org/10.1186/1471-244X-14-143
Frank, F., Wilk, J., Kriston, L., Meister, R., Shimodera, S., Hesse, K., … Hölzel, L. P. (2015). Effectiveness of a brief psychoeducational group intervention for relatives on the course of disease in patients after inpatient depression treatment compared with treatment as usual - Study protocol of a multisite randomised controlled trial. *BMC Psychiatry*, *15*(1), 1–11. https://doi.org/10.1186/s12888-015-0633-4
Franke, G. H. (1992). Eine weitere Überprüfung der Symptom-Check-Liste (SCL-90-R) als Forschungsinstrument. *Diagnostica*, *38*, 160– 167.
Franke, G. H. (2000). *Brief Symtpom Inventory von L. R. Derogatis - Deutsche Version*. Göttingen: Beltz Test GmbH.
Franz, M., Meyer, T., & Gallhofer, B. (2003). Belastungen von Angehörigen schizophren und depressiv Erkrankter – eine repräsentative Erhebung. In J. M. Fegert & U. Ziegenhain (Eds.), *Hilfen für Alleinerziehende. Die Lebenssituation von Einelternfamilien in Deutschland* (pp. 215–229). Berlitz.
Freitag, S., Stolzenburg, S., Schomerus, G., & Schmidt, S. (2018). Depressionswissen – Deutsche Übersetzung und Testung der Depression Literacy Scale. *Psychiatrische Praxis*, *45*(08), 412–419. https://doi.org/10.1055/s-0043-119245
Gerste, B., & Roick, C. (2014). Prävalenz und Inzidenz sowie Versorgung depressiver Erkrankungen in Deutschland – Eine Analyse auf Basis der in Routinedaten dokumentierten Depressionsdiagnosen. In J. Klauber, C. Günster, B. Gerste, B.-P. Robra, & N. Schmacke (Eds.),

Versorgungs- Report 2013/2014 Schwerpunkt: Depression (pp. 21–54). Stuttgart: Schattauer.
Giesinger, J., Rumpold, M. G., & Schüßler, G. (2008). Die K10-Screening-Skala für unspezifischen psychischen Distress. *Psychosomatik Und Konsiliarpsychiatrie*, *2*(2), 104–111. https://doi.org/10.1007/s11800-008-0100-x
Gräfe, K., Zipfel, S., Herzog, W., & Löwe, B. (2004). Screening psychischer Störungen mit dem "Gesundheitsfragebogen für Patienten (PHQ-D)". *Diagnostica*, *50*(4), 171–181. https://doi.org/10.1026/0012-1924.50.4.171
Griffiths, K.M., Christensen, H., Jorm, A.F., Evans, K., Groves, C. (2004). Effect of web-based depression literacy and cognitive-behavioural therapy interventions on stigmatising attitudes to depression: Randomised controlled trial. *The British Journal of Psychiatry*, *185*, 342–349.
Guizzaro, L., Pétavy, F., Ristl, R., & Gallo, C. (2020). The use of a variable representing compliance improves accuracy of estimation of the effect of treatment allocation regardless of discontinuation in trials with incomplete follow-up. *Statistics in Biopharmaceutical Research*, 1–18. https://doi.org/10.1080/19466315.2020.1736141
Hölzel, L., Härter, M., Reese, C., & Kriston, L. (2011). Risk factors for chronic depression — A systematic review. *Journal of Affective Disorders*, *129*(1–3), 1–13. https://doi.org/10.1016/j.jad.2010.03.025
Idstad, M., Ask, H., & Tambs, K. (2010). Mental disorder and caregiver burden in spouses: the Nord-Trøndelag health study. *BMC Public Health*, *10*(1), 516. https://doi.org/10.1186/1471-2458-10-516
Jacob, G., & Bengel, J. (2000). Das Konstrukt Patientenzufriedenheit: Eine kritische Bestandsaufnahme. *Zeitschrift Für Klinische Psychologie, Psychiatrie Und Psychotherapie*, *48*, 280–301.
Jacobi, F., Höfler, M., Strehle, J., Mack, S., Gerschler, A., Scholl, L., … Wittchen, H.-U. (2014). Psychische Störungen in der Allgemeinbevölkerung. Studie zur Gesundheit Erwachsener in Deutschland und ihr Zusatzmodul Psychische Gesundheit (DEGS1-MH). *Der Nervenarzt*, *85*(1), 77–87. https://doi.org/10.1007/s00115-013-3961-y
Katsuki, F., Takeuchi, H., Konishi, M., Sasaki, M., Murase, Y., Naito, A., … Furukawa, T. A. (2011). Pre-post changes in psychosocial functioning among relatives of patients with depressive disorders after Brief Multifamily Psychoeducation: A pilot study. *BMC Psychiatry*, *11*. https://doi.org/10.1186/1471-244X-11-56
Kelders, S. M., Bohlmeijer, E. T., Pots, W. T. M., & van Gemert-Pijnen, J. E. W. C. (2015). Comparing human and automated support for depression: Fractional factorial randomized controlled trial. *Behaviour Research and Therapy*, *72*, 72–80. https://doi.org/10.1016/j.brat.2015.06.014
Kessler, R. C., Andrews, G., Colpe, L. J., Hiripi, E., Mroczek, D. K., Normand, S.-L. T., … Zaslavsky, A. M. (2002). Short screening scales to monitor population prevalences and trends in non-specific psychological distress. *Psychological Medicine*, *32*(6), 959–976. https://doi.org/10.1017/S0033291702006074
Klaghofer, R., Brähler, E. (2001). Konstruktion und Teststatistische Prüfung einer Kurzform der SCL-90–R [Construction and test statistical evaluation of a short version of the SCL-90–R]. *Zeitschrift Für Klinische Psychologie, Psychiatrie Und Psychotherapie*, *49(2)*, 115–124.
König, H.-H., Luppa, M., & Riedel-Heller, S. (2010). Die Kosten der Depression und die Wirtschaftlichkeit ihrer Behandlung. *Psychiatrische Praxis*, *37*(05), 213–215. https://doi.org/10.1055/s-0030-1248510
Laux, L., Glanzmann, P., Schaffner, P., Spielberger, C. D. (1981). *Das State-Trait-Angstinventar*. Göttingen: Beltz Test GmbH.
Leithäuser, T., & Volmerg, B. (1988). *Psychoanalyse in der Sozialforschung*. Wiesbaden: VS Verlag für Sozialwissenschaften. https://doi.org/10.1007/978-3-663-07773-2
Löwe, B., Spitzer, R.L., Zipfel, S., Herzog, W. (2002). *Gesundheitsfragebogen für Patienten (PHQ D). Komplettversion und Kurzform. Testmappe mit Manual, Fragebögen, Schablonen* (2nd ed.). Karlsruhe: Pfitzer.
Meister, R., Jansen, A., Berger, M., Baumeister, H., Bschor, T., Harfst, T., … Härter, M. (2018). Psychotherapie depressiver Störungen: Verfahren, Evidenz und Perspektiven. *Der Nervenarzt*,

89(3), 241–251. https://doi.org/10.1007/s00115-018-0484-6
Perlick, D. A., Rosenheck, R. R., Clarkin, J. F., Raue, P., & Sirey, J. (2001). Impact of family burden and patient symptom status on clinical outcome in bipolar affective disorder. *The Journal of Nervous and Mental Disease*, *189*(1), 31–37.
Petrowski, K., Schmalbach, B., Kliem, S., Hinz, A., & Brähler, E. (2019). Symptom-Checklist-K-9: Norm values and factorial structure in a representative German sample. *PLOS ONE*, *14*(4), e0213490. https://doi.org/10.1371/journal.pone.0213490
Polverejan, E., & Dragalin, V. (2020). Aligning treatment policy estimands and estimators—a simulation study in Alzheimer's disease. *Statistics in Biopharmaceutical Research*, *12*(2), 142–154. https://doi.org/10.1080/19466315.2019.1689845
Robert Koch-Institut. (2018). Gesundheitsberichterstattung des Bundes - Verlorene Erwerbstätigkeitsjahre in 1.000 Jahren für Deutschland. Retrieved from http://www.gbe-bund.de/oowa921-install/servlet/oowa/aw92/WS0100/_XWD_PROC?_XWD_374/2/XWD_CUBE.DRILL/_XWD_402/D.946/14321
Rummel-Kluge, C., Kluge, M., & Kissling, W. (2015). Psychoedukation bei Depression: Ergebnisse zweier Umfragen im Abstand von fünf Jahren in Deutschland, Österreich und der Schweiz. *Psychiatrische Praxis*, *42*(6), 309–312. https://doi.org/10.1055/s-0035-1552662
Schulz, K. F., Altman, D. G., & Moher, D. (2010). CONSORT 2010 Statement: updated guidelines for reporting parallel group randomised trials. *BMJ*, *340*, c332. https://doi.org/10.1136/bmj.c332
Shimazu, K., Shimodera, S., Mino, Y., Nishida, A., Kamimura, N., Sawada, K., … Inoue, S. (2011). Family psychoeducation for major depression: Randomised controlled trial. *British Journal of Psychiatry*, *198*(5), 385–390. https://doi.org/10.1192/bjp.bp.110.078626
Spitzer, R. L. (1999). Validation and Utility of a Self-report Version of PRIME-MD<SUBTITLE>The PHQ Primary Care Study</SUBTITLE>. *JAMA*, *282*(18), 1737. https://doi.org/10.1001/jama.282.18.1737
Steele, A., Maruyama, N., & Galynker, I. (2010). Psychiatric symptoms in caregivers of patients with bipolar disorder: A review. *Journal of Affective Disorders*, *121*(1–2), 10–21. https://doi.org/10.1016/j.jad.2009.04.020
van Wijngaarden, B., Schene, A. H., Koeter, M., Vázquez-Barquero, J. L., Knudsen, H. C., Lasalvia, A., & McCrone, P. (2000). Caregiving in schizophrenia: development, internal consistency and reliability of the Involvement Evaluation Questionnaire – European Version. *British Journal of Psychiatry*, *177*(S39), s21–s27. https://doi.org/10.1192/bjp.177.39.s21
van Winjgaarden, B. S. A. H. (1992). *The Involvement Evaluation Questionnaire*. Amsterdam: Department of Psychiatry, University of Amsterdam.
Vos, T., Flaxman, A. D., Naghavi, M., Lozano, R., Michaud, C., Ezzati, M., … Murray, C. J. L. (2012). Years lived with disability (YLDs) for 1160 sequelae of 289 diseases and injuries 1990-2010: A systematic analysis for the Global Burden of Disease Study 2010. *The Lancet*, *380*(9859), 2163–2196. https://doi.org/10.1016/S0140-6736(12)61729-2
WHO. (1998). *Wellbeing Measures in Primary Healh Care/The DEPCARE Project*. Stockholm.
Wiedemann, G., Rayki, O., Feinstein, E., & Hahlweg, K. (2002). The Family Questionnaire: Development and validation of a new self-report scale for assessing expressed emotion. *Psychiatry Research*, *109*(3), 265–279. https://doi.org/10.1016/S0165-1781(02)00023-9
Wittmund, B., Wilms, H.-U., Mory, C., & Angermeyer, M. C. (2002). Depressive disorders in spouses of mentally ill patients. *Social Psychiatry and Psychiatric Epidemiology*, *37*(4), 177–182. https://doi.org/10.1007/s001270200012
World Health Organization. (2017). *Depression and Other Common Mental Disorders: Global Health Estimates*. Geneva: World Health Organization.

Gemeinsam durch die Depression

Facing Depression Together:

Evaluation of an online self-help program with individualized vs automated supportive message system for relatives, significant others, and caregivers of depressed persons

Statistical Analysis Plan

Version 1.0

Date: 20/Mar/2023

Project-No.: DRKS00025241

Protocol version 2.1

Protocol date 11/Aug/2022

Signature Page and Approval

**Trial Statistician**	**Dr. Erika Graf**	
**Co-Author**	**Signature:**	**Date:** 08.05.2023
**Statistical Programmer**	**Dr. Jörg Sahlmann**	
**Co-Author**	**Signature:**	**Date:** 08.05.23
**Project Leader**	**Prof. Dr. Elisabeth Schramm**	
	**Accepted and approved.**<br>**Signature:**	**Date:** 28.04.'23
**Project Coordinator**	**Dipl. Psych. Dr. Christoph Breuninger**	
	**Accepted and approved.**<br>**Signature:**	**Date:** 20.4.23
**Project Coordinator**	**Nadine Zehender, M. Sc.**	
	**Accepted and approved.**<br>**Signature:** N. Zehender	**Date:** 28.04.'23

Signature Page and Approval

Trial Statistician<br>Co-Author	Dr. Erika Graf	
	Signature:	Date:
Statistical Programmer<br>Co-Author	Dr. Jörg Sahlmann	
	Signature:	Date:
Project Leader	Prof. Dr. Elisabeth Schramm	
	Accepted and approved.<br>Signature:	Date:
Project Coordinator	Dipl. Psych. Dr. Christoph Breuninger	
	Accepted and approved.<br>Signature:	Date:
Project Coordinator	Nadine Zehender, M. Sc.	
	Accepted and approved.<br>Signature:	Date:

Table of contents

1 Scope of this document ... 5
2 Study design ... 6
3 Study objectives and endpoints ... 7
3.1 Study objectives and hypotheses ... 7
3.1.1 Primary hypotheses ... 7
3.1.2 Secondary hypotheses and goals ... 7
3.2 Study endpoints ... 7
3.2.1 Primary endpoint ... 7
3.2.2 Secondary endpoints ... 8
4 Interim analysis ... 9
5 Analysis sets ... 10
5.1 Definition of full analysis set ... 10
6 Statistical methods ... 11
6.1 Software ... 11
6.2 Data listings ... 11
6.3 Descriptive statistics ... 11
6.4 Data handling ... 11
6.4.1 Missing values ... 11
6.4.2 Outliers ... 11
6.4.3 Further details and conventions ... 12
6.4.4 Coding ... 12
7 Study caregivers and depressed persons ... 13
7.1 Disposition of caregivers and depressed persons ... 13
7.2 Screening failures ... 13
7.3 Protocol deviations ... 13
7.4 Compliance with planned visits ... 14
7.4.1 Caregivers ... 14
7.4.2 Depressed persons ... 14
7.5 Baseline characteristics of caregivers and depressed persons ... 14
7.6 Adherence to the online self-help program for caregivers ... 15
7.6.1 Online self-help program with individual support (IND) ... 15
7.6.2 Online self-help program with automated support (AUT) ... 15
7.6.3 Treatment as usual control condition with written information (TAU) ... 15
8 Efficacy evaluation ... 17
8.1 Analysis of primary endpoint ... 17

8.1.1 Primary efficacy analysis 17
8.1.2 Sensitivity analysis of primary outcome 18
8.1.3 Supplementary analysis of primary outcome scale K-10 19
8.2 Analyses of secondary endpoints 20
9 Safety evaluation 21
9.1 Adverse events questionnaire 21
9.2 Serious adverse events 21
10 History of changes 22
10.1 Changes to the clinical trial protocol (CTP) 22
10.2 Changes to the previous version of this SAP 22
10.3 Changes in this SAP compared to the CTP 22
11 List of abbreviations 23
12 Appendix 24
12.1 List of tables 24
12.1.1 Disposition of caregivers and protocol deviations 24
12.1.2 Baseline characteristics of caregivers and depressed persons 24
12.1.3 Adherence to the online self-program for caregivers 24
12.1.4 Efficacy data 25
12.1.5 Safety data 27
12.2 List of figures 27
12.2.1 Study caregivers and depressed persons 27
12.3 List of listings 28
12.3.1 Disposition of caregivers and protocol deviations 28
12.3.2 Baseline characteristics of caregivers and depressed persons 28
12.3.3 Safety data 28
13 Literaturverzeichnis 30

1 Scope of this document

This statistical analysis plan (SAP) describes the objectives, analysis populations and statistical methods of the statistical analyses of this trial to be performed at the Institute of Medical Biometry and Statistics. Further analyses aimed at the identification of moderator and mediator variables as well as process and qualitative analyses, to be performed at the Department of Psychiatry and Psychotherapy, will be described elsewhere.

The contents of this document are based on chapter 6 of the study protocol version 1.0 and its amended versions 2.0 and 2.1, approved by the Ethics Committee of the University of Freiburg on February 11, 2021, November 16, 2021, and August 11, 2022, respectively.

2 Study design

This is a randomized controlled open-label superiority trial with three parallel groups. Caregivers of depressed persons are randomized to one of three interventions: an online self-help program with individual support (IND), an online self-help program with automated support (AUT), and a control condition with written information, treatment as usual (TAU).

Randomization is performed as block randomization with a 2:2:1 allocation ratio (IND:AUT:TAU), with stratification based on the participating caregivers' characteristics as follows:

1. K-10-scale (10-22, 23-50, see endpoints, questionnaire and evaluation see Kessler (2001) and Kessler et al. (2002))
2. Gender (female, male; caregivers of diverse gender are assigned alternatingly to the groups of females and males)
3. Relation with depressed person (parent, child, partner, other)
4. Age (completed years, 18-40, 41-65, ≥ 66)

After informed consent and screening, time points of measurements are prior to randomization at pre-intervention/baseline (T0), one week (T1, only K-10-scale), four weeks (T2, end of intervention period), and three months after randomization (T3, follow-up), respectively, as shown in the following figure.

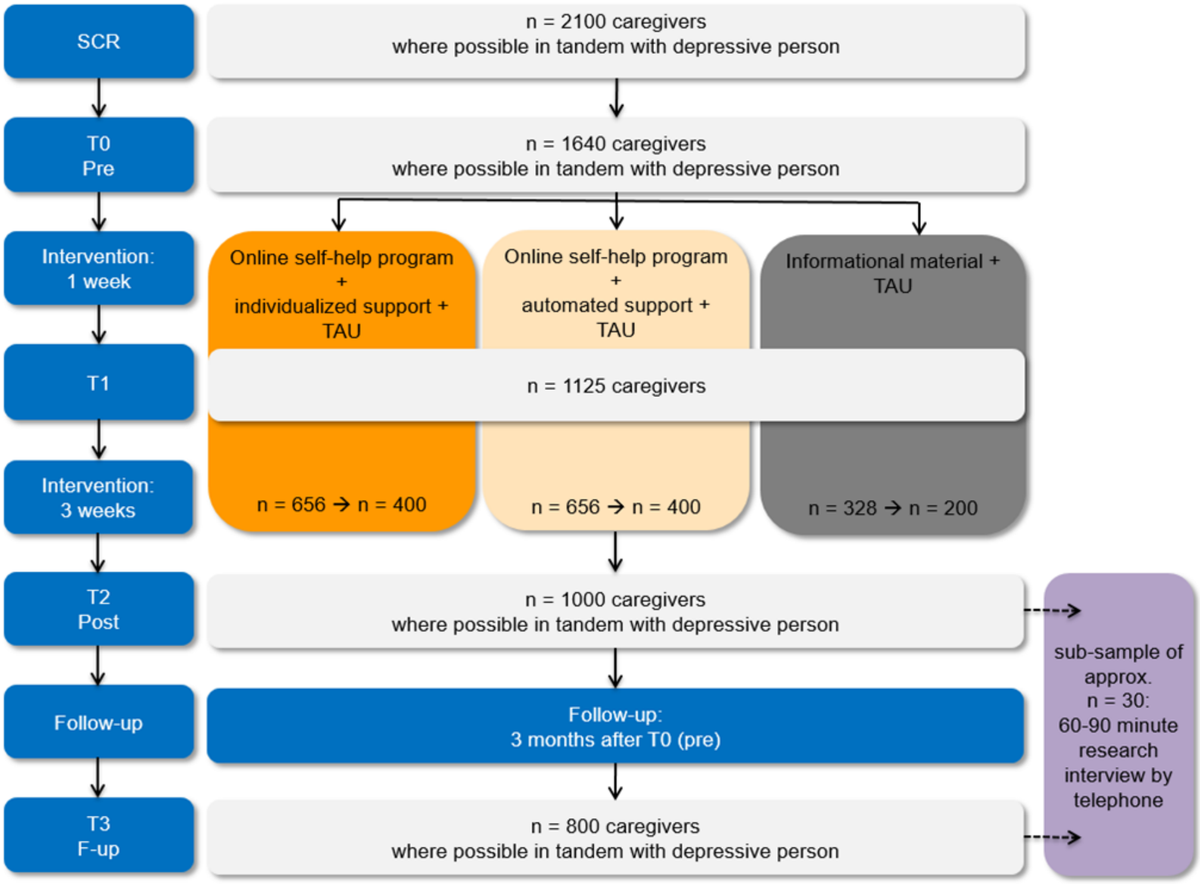

3 Study objectives and endpoints

3.1 Study objectives and hypotheses

Study objectives, estimand and hypotheses according to study protocol sections 3.2 and 3.3 are as follows.

With this trial it is intended to increase the efficacy of an online self-help program for caregivers of depressed persons by developing and implementing individualized vs automated support via secure e-mail messages.

The following primary research question will be addressed: Which impact has the four-week usage of the online self-help program with individualized or automated e-mail support vs treatment as usual on the risk of mental diseases in caregivers?

In order to achieve this, the study aims to determine if a treatment policy assigning caregivers of depressed persons to an individualised (IND) or automated (AUT) online self-help intervention reduces the caregiver's unspecific mental distress compared to assignment to treatment as usual (TAU), as measured by the change of the K-10 score from baseline to four weeks after randomisation (post-measurement minus baseline).

The change in mental distress is relevant regardless of whether or not the caregiver adheres to or discontinues the assigned treatment. Treatment arms should be compared with respect to the difference in means of the change in K-10 score (estimand).

3.1.1 Primary hypotheses

The online self-help program with individualized (IND) or automated (AUT) e-mail support reduces the unspecific mental distress of the caregivers (as an indicator for the risk of mental diseases) as compared to TAU. In a comparison of the two kinds of support, the individualized support will be more effective than the automated support.

3.1.2 Secondary hypotheses and goals

- The online self-help program with individualized or automated e-mail support reduces the psychosocial burden and the subjective symptom burden of the caregivers as well as the depressive symptoms in the depressed person. It improves depression literacy, interaction behaviour and the well-being of the caregivers.
- Assessing the acceptance and usage of the online self-help program
- Identification of moderators and mediator variables (to be described elsewhere)
- Qualitative evaluation (to be described elsewhere)

3.2 Study endpoints

The endpoints described in the study protocol in section 4.4 are as follows.

3.2.1 Primary endpoint

The primary outcome of the study is the unspecific psychological distress as indicator for the risk of mental illness in caregivers (K-10-scale) at post-intervention (T2), measured by the change of the K-

10 score from baseline (T0) to four weeks after randomization (T2, post-measurement minus baseline).

3.2.2 Secondary endpoints

All outcomes scales are to be assessed at pre-intervention (T0), post-intervention (T2) and at 3-month follow-up (T3). Secondary endpoints are assessed at T2 and T3.

3.2.2.1 For caregivers

- The subjective und objective psychosocial burden of caregiving (involvement evaluation questionnaire / IEQ-EU)
- The subjective symptom burden (symptom checklist short version / SCL-K-9)
- The depression literacy (depression literacy test; D-Lit)
- High expressed emotion aspects “critical comments” and
- “over-involvement” (Familienfragebogen; FFB)
- The well-being (WHO-5)
- Acceptance, adherence and usages questionnaire

3.2.2.2 For depressed persons

- Depressive symptoms (patient health questionnaire PHQ-9).

Calculation of scores will be done according to the respective manual.

4 Interim analysis

No interim analyses will be performed, according to study protocol sections 6.3.2, 7.1 and 7.2.

5 Analysis sets

All statistical analyses will be performed in the full analysis set (FAS). Restrictions to subpopulations of the FAS (e.g. caregivers with specific missing or non-missing outcomes) will be described in the subsequent text, tables, figures and data listings as appropriate.

5.1 Definition of full analysis set

The FAS includes all randomized caregivers. Caregivers are analysed as belonging to their randomized arm (IND, AUT, and TAU) regardless of whether they refused or discontinued therapy, or whether other protocol deviations are known.

The analysis of the primary endpoint will be done according to the intention-to-treat (ITT) principle, i.e. based on the 'full analysis set', to assess how a treatment policy assigning caregivers of depressed persons to IND, AUT or TAU affects the caregivers' change in mental distress. Treatment arms should be compared with respect to the difference in means of the change in K-10 score (estimand).

6 Statistical methods

The statistical methods described in this SAP are in accordance with the analysis planned in the study protocol.

6.1 Software

Statistical computing will be performed with R (Version 4.1.2 or higher) or with the Statistical Analysis System (SAS).

6.2 Data listings

Data listings will be provided as specified. Upon request, SDTM data sets can be provided in a filterable and searchable manner (xlsx file).

Variables given as free text (such as “other, please specify”) will be listed.

6.3 Descriptive statistics

In general, continuous data will be summarised by arithmetic mean, standard deviation, minimum, 25% quantile, median, 75% quantile, maximum, number of complete observations, and absolute and relative numbers of non-missing observations. If appropriate, continuous variables can also be presented in categories.

In general, categorical data will be summarised by the total number of caregivers in each category and the number of missing values. Relative frequencies are displayed as valid “%” (100 times the number of caregivers divided by the number of caregivers with non-missing values).

Depending on the kind of the table, the selection of the displayed statistics might change.

Data items needed for reporting of trial results in compliance with the CONSORT (Schulz et al. (2010)) and CONSORT-EHEALTH (Eysenbach (2011)) statements will be evaluated. They are included in the tables and figures described in section 7.

6.4 Data handling

6.4.1 Missing values

In general, missing values will not be replaced. Handling of missing values for the primary endpoint: Multiple imputation will be employed to replace missing values in the primary analysis (more details in section 8.1).

Missing items in the score questionnaires will be treated as specified in the respective manual.

6.4.2 Outliers

No formal analyses for outliers will be done. No participants will be excluded. Should the presence of observed outliers cast doubts upon the validity of a specific analysis result, the analysis with the actual values will be supplemented by a post-hoc analysis eliminating or reducing the outlier effect.

6.4.3 Further details and conventions

No further conventions.

6.4.4 Coding

No formal systems for coding the data such as MedDRA or WHO-ATC will be used. Variables given as free text (such as “other, please specify”) can be summarized by groups as deemed appropriate based on the answers given. Such categorization will be done by the study coordinators and provided to the biostatistician.

7 Study caregivers and depressed persons

7.1 Disposition of caregivers and depressed persons

Recruitment of randomized caregivers and depressed persons over time will be graphically displayed for the FAS not separated by intervention (figure B1-1).

The numbers of caregivers who were randomized, and who entered and completed each assessment period (questionnaires at T0, T1 (only K-10), T2 and T3) of the study should be provided. Entering an assessment period means that at least one item of the respective phase is provided. Completing an assessment period means that the caregiver or depressed person explicitly confirms having finished the respective period. The number of caregivers in the FAS will be given in total and by randomized intervention (table A1-1).

A flow chart according to the CONSORT statement will be given (figure B1-2). The flow chart will present an overview of the number of caregivers for each phase of the trial:

- Informed consent, assessed for eligibility, excluded (with reasons), randomized
- Started allocated intervention, did not start allocated intervention (by group; IND, AUT: Percent total progress in coach (= cumulative percent of pages in coach read by caregiver in week 4) >0 or =0; TAU: all randomized caregivers start allocated intervention)
- Minimal use: Percent total progress in coach < 10% (applicable for IND and AUT)
- Change of K-10 score from baseline to T2 missing (by group)
- Analysed in primary analysis (by group)
- Completed assessment period T3 (by group)

Percent total progress in coach will be categorized:

- 0% ≤ x < 10%: minimal use
- 10% ≤ x < 25%: limited use
- 25% ≤ x: intended use

In tabular overviews, the first and last dates of informed consent, first login, last login, first T0 answer, completing T0 answer (randomisation), first T3 answer and completing T3 answer (end of study) will be given along with absolute numbers of caregivers (table A1-2) and depressed persons (table A1-3) per randomised intervention and in total. The individual data will be listed in C1-1.

7.2 Screening failures

If any data for screening failure is available, the screening failures will be displayed in a summary table for all caregivers and depressed persons, respectively (table A1-4). Screening failures are persons who gave informed consent, but were not randomised.

7.3 Protocol deviations

Not applicable

Reason: Only eligible caregivers are randomised, based on the caregivers' answers to the questionnaire concerning inclusion and exclusion criteria. Post-randomisation protocol deviations are not applicable since the protocol does not contain any prescriptions for caregivers.

7.4 Compliance with planned visits

The following information for the compliance with planned visits will be given in table A1-5 for caregivers and in table A1-6 for depressed persons:

7.4.1 Caregivers

- Number of caregivers for whom a visit was completed (possible time interval for performing the questionnaires: 2 weeks for T0, 1 week for T1, 4 weeks for T2, 4 weeks for T3)
- Percentages of caregivers for whom a visit was completed (for visits T1, T2 and T3 with reference to the number of caregivers randomized after completion of T0)
- Distribution of the actual time intervals from T0 to T1, T2 and T3
- Difference between actual and planned time intervals from T0 to T1, T2 and T3
- Number of visits missing

7.4.2 Depressed persons

- Number of depressed persons for whom a visit was completed (released time interval for performing the questionnaires: 4 weeks for T0, 2 months for T2, 4 weeks for T3)
- Percentages of caregivers for whom a visit was completed (for visits T2 and T3 with reference to the number of caregivers randomized after completion of T0)
- Distribution of the actual time intervals from T0 to T2 and T3
- Difference between actual and planned time intervals from T0 to T2 and T3
- Number of visits missing

7.5 Baseline characteristics of caregivers and depressed persons

Demographic and other baseline characteristics will be listed per caregiver and depressed person as well as summarized in total and by randomized intervention in the FAS (listing C2-1, table A2-1).

These factors include following sociodemographic variables of the caregivers and depressed persons:

- Age (completed years, continuous and categorized for analysis: 18-40, 41-65, ≥ 66)
- Gender (female, male, diverse)
- Highest educational qualification (German Hauptschulabschluss, German Mittlere Reife, Abitur, no educational qualification)
- Current job situation (student, employed, unemployed, housewife/house husband, retired)
- Caregiver’s relation with depressed person (parent, child, partner, other)
- Common household with the depressed person (yes, no)
- Change of the job situation due to the depression of the relative (yes (reduction), yes (increase), no)
- Official diagnosis of the depression of the relative (yes, no with explanation)
- In case of an existing treatment of the depression: description
- First two numbers of the PLZ (postal ZIP code)
- Score of internet literacy

7.6 Adherence to the online self-help program for caregivers

The online self-help program consists of four interactive, independently usable modules. During the study period of four weeks, the online self-program varies depending on the randomized intervention.

7.6.1 Online self-help program with individual support (IND)

The following information on caregiver's accessing of the modules of the online-self program in the framework of IND will be summarized (table A3-1):

- Number of sessions by caregiver: in total, in week 1, 2, 3 and 4
- Number of active days by caregiver: in total, in week 1, 2, 3 and 4
- Duration of time spent by caregiver: in total, in week 1, 2, 3 and 4
- Percent progress in coach (= cumulative percent of pages in coach read by caregiver): in week 1, 2, 3 and 4 (= total)
- Number of messages from psychologist to caregiver: in total, in week 1, 2, 3 and 4
- Number of messages from caregiver to psychologist: in total, in week 1, 2, 3 and 4

Caregivers with intervention IND will be considered as adherent if their total duration of time spent up to week 4 (T2) is more than 1.5 hours and their percent total progress in coach is more than 25%. Number and percentages of adherent caregivers will be shown in table A3-4 by intervention.

7.6.2 Online self-help program with automated support (AUT)

The following information on completing the modules of the online self-help program in the framework of AUT will be summarized (table A3-2):

- Number of sessions by caregiver: in total, in week 1, 2, 3 and 4
- Number of active days by caregiver: in total, in week 1, 2, 3 and 4
- Duration of time spent by caregiver: in total, in week 1, 2, 3 and 4
- Percent progress in coach (= cumulative percent of pages in coach read by caregiver): in week 1, 2, 3 and 4 (= total)

Like caregivers with intervention IND, caregivers with intervention AUT will be considered as adherent if their total duration of time spent up to week 4 (T2) is more than 1.5 hours and their percent total progress in coach is more than 25%. Number and percentages of adherent caregivers will be shown in table A3-4 by intervention.

7.6.3 Treatment as usual control condition with written information (TAU)

The following information within the framework of TAU for caregiver will be summarized (table A3-3):

- Number of sessions by caregiver: in total, in week 1, 2, 3 and 4
- Number of active days by caregiver: in total, in week 1, 2, 3 and 4
- Duration of time spent by caregiver: in total, in week 1, 2, 3 and 4

Caregivers with intervention TAU will always be considered as adherent because the variable checking whether the document of instructions for caregivers in TAU was downloaded was introduced while the study already running. These download frequencies will be reported descriptively (table A3-5).

8 Efficacy evaluation

Throughout, emphasis will be given to reporting of effect estimators with two-sided 95%-confidence intervals rather than p-values whenever possible, which will be considered statistically significant if below 5% for tests of null hypothesis of equality. However, confirmatory statistical testing will be restricted to the analysis of the primary outcome, to be followed by descriptive reporting of other trial results.

8.1 Analysis of primary endpoint

8.1.1 Primary efficacy analysis

The primary efficacy analysis will be performed in the full analysis set (FAS) according to the intention-to-treat (ITT) principle. Therefore, all randomized caregivers will be analysed in the assigned intervention arms, irrespective of intervention adherence and whether they refuse or discontinue the intervention. The effects of allocation to IND, AUT and TAU with respect to the primary endpoint, change in K-10 score from baseline to four weeks after randomisation, will be estimated and tested in a linear regression model including multiply imputed missing values calculated and combined as described below, and the corresponding two-sided 95%-confidence intervals will be reported (table A4-1). The model will include randomized intervention (IND, AUT, TAU), age (years, 18-40, 41-65, ≥ 66), gender (female, male; for diverse, see end of paragraph), caregiver's relation with depressed person (parent, child, partner, other), and continuous K-10 baseline score as independent variables. Since very few caregivers of diverse gender are expected, they will be assigned alternatingly to the groups of females and males in the primary analysis of intervention effects. They will be analysed separately in secondary analyses exploring the effect of gender (section 8.1.3).

Sequential testing: Following a sequential closed testing procedure to ensure a multiple type I error rate of 5%, confirmatory comparisons of randomized intervention arms will proceed until the first occurrence of a non-significant result at the nominal 5% level, followed by descriptive reporting of all subsequent analyses. The two-sided tests of equality of means of two intervention arms will be based on the two-sided 95% confidence interval for the difference in mean change from baseline estimated from the linear regression model. Intervention arms will be compared in the following order:

1. IND versus TAU
2. AUT versus TAU
3. IND versus AUT

The results of this sequential testing will be displayed in table A4-2.

Although it is hypothesized and subjected to confirmatory testing that IND is superior to AUT, equality is also deemed possible. In a secondary descriptive analysis, demonstration of non-inferiority of AUT compared to IND will also be attempted. If the lower limit of the two-sided 95%-confidence interval for the difference of mean K-10 changes from baseline (IND minus AUT) is greater than -0.62 points, this will be viewed as evidence for non-inferiority (table A4-3). The non-inferiority margin was derived as follows: For caregivers of depressed persons, the expected standard deviation of K-10 scores is in between that of caregivers (SD: 4.6 points) and students (SD: 7.8 points) seen in the German norm sample (Giesinger et al. (2008)). Therefore, a standard deviation of 6.2 score points is expected. Assuming a correlation of 0.5 between baseline and post-intervention K-10 scores, the change from baseline has also a standard deviation of 6.2 points, and using Cohen's d=0.1 as non-inferiority margin gives 0.62 points.

Handling of missing values: Multiple imputation will be employed to replace missing values. Imputation will be done per randomized intervention arm, using baseline data and post-baseline information according to a Treatment Policy Strategy as described by Polverejan und Dragalin (2020) and Guizzaro et al. (2021). Imputed values that fall below or above the range of values of the K-10 score will be set to the corresponding lower or upper limit. The results will be combined using Rubin's rules for multiple imputation (Rubin (1987)).

- Missing post-baseline values of K-10 scores will be imputed using a fully conditional specification in separate linear models for each of the respective randomized arms TAU, AUT and IND, with different sets of independent variables as follows.
- For TAU, AUT and IND, the model will include age group, gender, caregiver's relation with depressed person, continuous baseline and post-baseline K-10 measurements as well as continuous baseline internet literacy scores as independent variables. K-10 scores will be imputed in the following order: T1, T3, T2.
- For TAU, data will be imputed from this model.
- For AUT and IND, the caregiver's total duration of time (> 1.5 hours) spent up to week 4 (T2) and the percent total progress in coach (rate p, logit-transformed as follows: (p+0.01)/(1.01-p)) will be included as additional independent variables.
- For IND, the cumulative number of messages from caregiver to psychologist at T2 (after Box-Cox transformation) will be modelled as additional continuous independent variable.
- Number of imputations and random seed: The number of imputations is set to 10.000. The random seed for imputation will be set to the date one day after data base lock in the format yyyymmdd (year-month-day).

Assumptions: The resulting estimators assume that caregivers with missing outcome data have outcomes similar to those of subjects with similar baseline data and intervention adherence who provide their outcome data, i.e., outcome data at T2 are assumed to be missing at random given the independent variables.

Additional output

The results of multiple imputation with respect to change in K-10 score from baseline to T1 and T3, combined using Rubin's rules for multiple imputation as described for the primary endpoint in table A4-1 will be shown in tables A4-4 (T1) and A4-5 (T3).

Descriptive summaries for the outcome scale of the primary endpoint will be given in tables (K-10 score at T0, T1, T2, and T3: table A4-6, change of K-10 score from baseline to T1, T2, T3: table A4-7).

Least-squares means for K-10 scores per randomized arm at T0, T1, T2, T3 calculated from these models will be shown with two-sided 95%-confidence intervals in figure B4-1. At T0, the identical mean and standard deviation pooled across all randomized caregivers (not separated by arm) will be used for the calculation within arms. At T1, T2 and T3, least-squares means per randomized arms will be calculated at the observed T0 all-group means of the other independent variables.

8.1.2 Sensitivity analysis of primary outcome

To evaluate the robustness of the main analysis with respect to the assumptions on missingness, an alternative multiple imputation model will be applied for sensitivity analysis. It will impute the outcomes that are missing such that outcomes in the IND and AUT arms are assumed to be similar to those observed in the TAU group, based on the imputation model for TAU of the primary analysis. The results of the sensitivity analysis with respect to change in K-10 score from baseline to T2

assuming missing values to be similar to those observed in the TAU group will be shown in table A4-8.

To evaluate the robustness of the comparison of IND versus AUT in the main analysis with respect to adherence, the primary efficacy analysis will be repeated in subpopulations of adherent AUT and IND caregivers as defined in section 7.6. If the lower limit of the two-sided 95%-confidence interval for the difference of mean K-10 changes from baseline (IND minus AUT) is greater than -0.62 points, this will be viewed as evidence for non-inferiority. The results of the sensitivity analysis with respect to change in K-10 score from baseline to T2 comparing AUT and IND in the subgroups of adherent caregivers will be shown in table A4-9.

8.1.3 Supplementary analysis of primary outcome scale K-10

In further exploratory analyses, the effects of randomized intervention on change of K-10 score from baseline will be evaluated without imputation of missing values in a longitudinal linear ANCOVA model accounting for correlations between T1-, T2- and T3-outcomes of the same caregiver. Independent variables will be age group, gender and caregiver's relation with depressed person as well as variables modelling time-dependent effects of randomized intervention and of continuous K-10 baseline score at T1, T2 and T3. The covariance structure will be initially specified as unstructured. If the model fails to converge, then a heterogeneous first-order autoregressive (ARH(1)) structure will be used, followed by a first-order autoregressive (AR(1)) structure. If these models fail to converge, the effect of the continuous K-10 baseline score will be modelled as a single, non-time-dependent covariate. Results will be presented in table A4-10.

Least-squares means for K-10 scores per randomized arm at T0, T1, T2, T3 calculated from this model will be shown with two-sided 95%-confidence intervals in figure B4-2. At T0, the identical mean and standard deviation pooled across all randomized caregivers (not separated by arm) will be used for the calculation within arms. At T1, T2 and T3, least-squares means per randomized arms will be calculated at the observed T0 all-group means of the other independent variables.

In order to assess the consistency of the intervention effect in relevant subgroups (EMA-CHMP, 2019), exploratory analyses will further expand the longitudinal ANCOVA model. Factors to be explored comprise all variables for grouping that were used for stratified randomization (i.e. age group, gender [female, male], relation with depressed person, K-10 score group at T0) as well as internet literacy score (two groups split at baseline median in all randomized caregivers). For each of these variables, respectively, time-dependent intervention effects within subgroups will be modelled by the corresponding interaction terms (group variable x intervention x time point) in a correspondingly modified longitudinal ANCOVA model (Tables A4-11 to A4-15).

Based on the respective expanded longitudinal ANCOVA model per subgroup variable, intervention effects (IND versus TAU, AUT versus TAU and IND versus AUT) will be estimated as difference in mean K-10 change from baseline to T2 with two-sided 95% confidence interval within each subgroup. Descriptive p-values to test for a difference of intervention effects between subgroups, derived from the T2 interaction terms of subgroup with intervention, will be given (Tables A4-16 to A4-20). The results shown in Tables A4-16 to A4-20 will be visualised in three figures showing subgroup-specific intervention comparisons at T2 for IND versus TAU, AUT versus TAU, and IND versus AUT, respectively (Figures B4-3 to B4-5). Due to the low percentage of caregivers with diverse gender, these will be excluded from the subgroup regression analysis of gender, and their outcomes per randomized arm will only be summarized descriptively.

Descriptive results per randomized intervention and time point will be given for all subgroup variables (K-10 score at T0, T1, T2, T3: Tables A4-20 to A4-24, change of K-10 score from baseline to T1, T2, T3: Tables A4-25 to A4-29).

8.2 Analyses of secondary endpoints

Secondary outcomes at T2, four weeks after randomization, and T3, three months after randomization, will be evaluated in a linear mixed model per main outcome scale, with a compound symmetry covariance matrix to account for correlation between T2- and T3-outcomes of the same caregiver or depressed person. Independent variables will be those of the main primary analysis, plus time point (T2, T3) and interaction terms between time point and randomized intervention (IND, AUT, TAU). (Tables IEQ-EU: A5-1, SCL-K-9: A5-3, D-Lit: A5-5, FFB: A5-7, WHO-5 A5-9, PHQ-9: A5-12).

Descriptive summaries for all secondary endpoints will be given (tables IEQ-EU: A5-2, SCL-K-9: A5-4, D-Lit: A4-6, FFB: A4-8, WHO-5: A4-10, acceptance, adherence and usages questionnaire: A5-11, PHQ-9: A5-13).

Least-squares means plots for all secondary endpoints will be given (figures IEQ-EU: B4-6, SCL-K-9: B4-7, D-Lit: B4-8, FFB: B4-9, WHO-5: B4-10, PHQ-9: B4-11).

Subgroup analyses, moderator and mediator analyses for the primary outcome will be performed outside the scope of the SAP, as described above.

Furthermore, the results seen in the Facing Depression Together trial will be compared to historical controls of face-to-face psychoeducation groups for caregivers of depressive in-caregivers from the multicentre SCHILD study (Frank et al. (2015)).

9 Safety evaluation

9.1 Adverse events questionnaire

The adverse events (AE) questionnaire for the caregivers, collected at T2 and T3, will be summarized in total and by randomized intervention in the FAS (table A6-1).

9.2 Serious adverse events

A serious adverse event (SAE) is any adverse event that:

- Results in death
- Is life threatening, or places the participant at immediate risk of death from the event as it occurred
- Requires or prolongs hospitalization
- Causes persistent or significant disability or incapacity
- Results in congenital anomalies or birth defects
- Is another condition which investigators judge to represent significant hazards

Each free text in the AE questionnaire is checked to see whether it contains an AE or a SAE. SAEs will be categorized according to the list of criteria mentioned in the previous paragraph.

Free texts containing an AE or a SAE will be listed in listing C3-2. Absolute frequencies in total and by randomized intervention will be provided in table A6-2.

Checking a Yes in one of the pre-specified items in the AE questionnaire does not imply a SAE according to the specifications.

10 History of changes

10.1 Changes to the clinical trial protocol (CTP)

CTP Version / Date / Section	Considered in SAP Version / Section	Description of changes	Reason for changes
2.0 / 29.10.2021	NA	Amendment	
1.0 / xx.11.2020	NA	First version	NA

10.2 Changes to the previous version of this SAP

SAP Version / Section	Description of changes	Reason for changes
NA	Amendment	
NA	First version	NA

10.3 Changes in this SAP compared to the CTP

SAP Version / Section	CTP Version / Section	Description of changes	Reason for changes
1.0 /	NA	Age in completed years	Exact specification
	Sensitivity analysis of primary outcome	…will impute the outcomes that are missing ~~due to study withdrawal~~	All missing values will be imputed. There are hardly any interim missing values.
	6.3.5. Analysis of secondary outcomes	No statistical comparison is made with the historical control group from the SCHILD study. If the results of the SCHILD study have already been published at the time of publication of the present study, they will be discussed.	Contrary to expectations, the data from the SCHILD study have not yet been published and therefore cannot be used and published in the context of the present study.

11 List of abbreviations

**Abbreviation**	**Abbreviated term**
AE	Adverse event
ANCOVA	Analysis of covariance
AR(1)	First-order autoregressive structure
ARH(1)	Heterogeneous first-order autoregressive structure
AUT	Online self-help program with automated support
CONSORT	Consolidated Standards of Reporting Trials
CRF	Case report form (= Prüfbogen bzw. Dokumentationsbogen, documentation sheet/form)
CTP	Clinical trial protocol
eCRF	electronic case report form
EMA-CHMP	European Medicines Agency – Committee for Medicinal Products for human use
FAS	Full analysis set
FFB	Familienfragebogen
IEQ-EU	Involvement Evaluation Questionnaire – European version
IND	Online self-help program with individual support
ITT	Intention-To-Treat
K-10	Kessler Psychological Distress Scale with 10 items
MedDRA	Medical Dictionary for Regulatory Affairs
PHQ-9	Patient Health Questionnaire with 9 items
SAE	Serious adverse event
SAP	Statistical analysis plan
SCL-K-9	Symptom Check List Short Version with 9 items
SDTM	Standard Data Tabulation Model
SOP	Standard operating procedure
TAU	Treatment as usual control condition with written information
WHO-ATC	World health organisation Anatomical Therapeutic Chemical
WHO-5	World health organisation Well-Being Index with 5 items

12 Appendix

12.1 List of tables

12.1.1 Disposition of caregivers and protocol deviations

Number	Title
A1-1	Number of caregivers in the FAS, in total and by randomized intervention
A1-2	Disposition of caregivers
A1-3	Disposition of depressed persons
A1-4	Screening failures
A1-5	Compliance with planned visits (caregivers)
A1-6	Compliance with planned visits (depressed persons)

12.1.2 Baseline characteristics of caregivers and depressed persons

Number	Title
A2-1	Demographics<br>(age, gender, highest educational qualification, current job situation, caregiver's relation with depressed person, common household with the depressed person, change of the job situation due to the depression of the relative, official diagnosis of the depression of the relative, existing treatment of the depression, first two numbers of the PLZ, score of internet literacy)

12.1.3 Adherence to the online self-program for caregivers

Number	Title
A3-1	Intervention arm IND<br>(Online self-help program in total, in week 1, 2, 3 and 4:<br>number of sessions, number of active days, duration of time spent, number of messages from psychologist to user, number of messages from user to psychologist)
A3-2	Intervention arm AUT<br>(Online self-help program in total, in week 1, 2, 3 and 4:<br>number of sessions, number of active days, duration of time spent)
A3-3	Intervention arm TAU<br>(Online self-help program in total, in week 1, 2, 3 and 4:<br>number of sessions, number of active days, duration of time spent)
A3-4	Number of adherent caregivers (intervention arms IND, AUT)<br><br>Frequencies and percentages of processing time greater than 1.5 hours<br>Frequencies and percentages of total progress greater than 25%<br>Cross tabulation<br><br>Each by intervention
A3-5	Download of instructions for intervention arm TAU

Number	Title

12.1.4 Efficacy data

12.1.4.1 Caregiver, primary outcome scale, multiple imputation for primary efficacy analysis

Number	Title
A4-1	Primary efficacy analysis – K-10 score, change from baseline to T2: Linear model after multiple imputation of missing values
A4-2	Sequential testing for comparison of intervention arms<br><br>1. IND versus TAU<br>2. AUT versus TAU<br>3. IND versus AUT
A4-3	Non-inferiority of interventions<br><br>AUT compared to IND
A4-4	K-10 score, change from baseline to T1: Linear model after multiple imputation of missing values
A4-5	K-10 score, change from baseline to T3: Linear model after multiple imputation of missing values

12.1.4.2 Descriptive summaries

Number	Title
A4-6	K-10 score: Descriptive summary at T0, T1, T2 and T3
A4-7	K-10 score, change from baseline: Descriptive summary at T1, T2 and T3

12.1.4.3 Sensitivity analyses

Number	Title
A4-8	K-10 score, change from baseline to T2: Sensitivity analysis, missing values imputed from TAU arm
A4-9	K-10 score, change from baseline to T2: Sensitivity analysis in subgroups of adherent AUT and IND caregivers, linear model after multiple imputation of missing values

12.1.4.4 Supplementary analyses, longitudinal ANCOVA model

Number	Title
A4-10	K-10 score, change from baseline: Longitudinal ANCOVA model

12.1.4.5 Supplementary analyses, longitudinal ANCOVA models including subgroup effects

Number	Title
A4-11	K-10 score, change from baseline: Longitudinal ANCOVA model including subgroup effects by age
A4-12	K-10 score, change from baseline: Longitudinal ANCOVA model including subgroup effects by gender
A4-13	K-10 score, change from baseline: Longitudinal ANCOVA model including subgroup effects by relation with depressed person
A4-14	K-10 score, change from baseline: Longitudinal ANCOVA model including subgroup effects by K-10 score at T0
A4-15	K-10 score, change from baseline: Longitudinal ANCOVA model including subgroup effects by internet literacy
A4-16	K-10 score, change from baseline: Longitudinal ANCOVA model, intervention effects at T2 by age group
A4-17	K-10 score, change from baseline: Longitudinal ANCOVA model, intervention effects at T2 by gender
A4-18	K-10 score, change from baseline: Longitudinal ANCOVA model, intervention effects at T2 by relation with depressed person
A4-19	K-10 score, change from baseline: Longitudinal ANCOVA model, intervention effects at T2 by K-10 score at T0
A4-20	K-10 score, change from baseline: Longitudinal ANCOVA model, intervention effects at T2 by internet literacy

12.1.4.6 Supplementary analyses, descriptive statistics by subgroup

Number	Title
A4-20	K-10 score: Descriptive summary at T0, T1, T2 and T3 by age group
A4-21	K-10 score: Descriptive summary at T0, T1, T2 and T3 by gender
A4-22	K-10 score: Descriptive summary at T0, T1, T2 and T3 by relation with depressed person
A4-23	K-10 score: Descriptive summary at T0, T1, T2 and T3 by K-10 score at T0
A4-24	K-10 score: Descriptive summary at T0, T1, T2 and T3 by internet literacy
A4-25	K-10 score, change from baseline: Descriptive summary at T1, T2 and T3 by age group
A4-26	K-10 score, change from baseline: Descriptive summary at T1, T2 and T3 by gender
A4-27	K-10 score, change from baseline: Descriptive summary at T1, T2 and T3 by relation with depressed person
A4-28	K-10 score, change from baseline: Descriptive summary at T1, T2 and T3 by K-10 score at T0
A4-29	K-10 score, change from baseline: Descriptive summary at T1, T2 and T3 by internet literacy

12.1.4.7 Caregivers, secondary outcome scales

Number	Title
A5-1	IEQ-EU score from baseline to T2 and T3: Linear mixed model (model includes: randomized intervention, gender, caregiver's relation with depressed person and IEQ-EU score at T0, T2 and T3)
A5-2	IEQ-EU: descriptive summary at T0, T2 and T3
A5-3	SCL-K-9 score from baseline to T2 and T3 : linear mixed model (model includes: randomized intervention, gender, caregiver's relation with depressed person and SCL-K-9 at T0, T2 and T3)
A5-4	SCL-K-9 : descriptive summary at T0, T2 and T3
A5-5	D-Lit from baseline to T2 and T3: Linear mixed model (model includes: randomized intervention, gender, caregiver's relation with depressed person and D-Lit at T0, T2 and T3)
A5-6	D-Lit: descriptive summary at T0, T2 and T3
A5-7	Secondary endpoint FFB from baseline to T2 and T3: Linear mixed model (model includes: randomized intervention, gender, caregiver's relation with depressed person and FFB at T0, T2 and T3)
A5-8	Secondary endpoint FFB: descriptive summary at T0, T2 and T3
A5-9	Secondary endpoint WHO-5 from baseline to T2 and T3: Linear mixed model (model includes: randomized intervention, gender, caregiver's relation with depressed person and WHO-5 at T0, T2 and T3)
A5-10	Secondary endpoint WHO-5: descriptive summary at T0, T2 and T3
A5-11	Secondary endpoint acceptance, adherence and usages questionnaire: descriptive summary at T2

12.1.4.8 Depressed person

Number	Title
A5-12	PHQ-9 from baseline to T2 and T3: Linear mixed model (model includes: randomized intervention, gender, caregiver's relation with depressed person and PhQ-9 at T0, T2 and T3)
A5-13	PHQ-9: descriptive summary at T0, T2 and T3

12.1.5 Safety data

Number	Title
A6-1	Adverse events questionnaire
A6-2	Serious adverse events questionnaire

12.2 List of figures

12.2.1 Study caregivers and depressed persons

Number	Title
B1-1	Recruitment of randomized caregivers and depressed persons over time
B1-2	CONSORT diagram

Number	Title
B4-1	K-10 score: Least-squares means, linear models after multiple imputation of missing values<br>(T0 over all; T1, T2, T3 by intervention)<br>T1, T2, T3 as dependent variable
B4-2	K-10 score: Least-squares means, longitudinal ANCOVA model
B4-3	K-10 score: Subgroup-specific intervention effects at T2 from longitudinal ANCOVA models , IND versus TAU<br>One figure per subgroup variable
B4-4	K-10 score: Subgroup-specific intervention effects at T2 from longitudinal ANCOVA models, AUT versus TAU<br>One figure per subgroup variable
B4-5	K-10 score: Subgroup-specific intervention effects at T2 from longitudinal ANCOVA models, IND versus AUT<br>One figure per subgroup variable
B4-6	Least-squares means plot IEQ-EU
B4-7	Least-squares means plot SCL-K-9
B4-8	Least-squares means plot D-Lit
B4-9	Least-squares means plot FFB
B4-10	Least-squares means plot WHO-5
B4-11	Least-squares means plot PHQ-9

12.3 List of listings

12.3.1 Disposition of caregivers and protocol deviations

Number	Title
C1-1	Disposition of caregivers
C1-2	Violation of inclusion criteria/ exclusion criteria

12.3.2 Baseline characteristics of caregivers and depressed persons

Number	Title
C2-1	Demographics<br>(age, gender, highest educational qualification, current job situation, caregiver's relation with depressed person, common household with the depressed person, change of the job situation due to the depression of the relative, official diagnosis of the depression of the relative, existing treatment of the depression, first two numbers of the PLZ)

12.3.3 Safety data

Number	Title
C3-1	Adverse events
C3-2	Serious adverse events

Number	Title

13 Literaturverzeichnis

Eysenbach, Gunther (2011): CONSORT-EHEALTH: improving and standardizing evaluation reports of Web-based and mobile health interventions. In: *Journal of medical Internet research* 13 (4), e126. DOI: 10.2196/jmir.1923.

Frank, Fabian; Wilk, Juliette; Kriston, Levente; Meister, Ramona; Shimodera, Shinji; Hesse, Klaus et al. (2015): Effectiveness of a brief psychoeducational group intervention for relatives on the course of disease in patients after inpatient depression treatment compared with treatment as usual--study protocol of a multisite randomised controlled trial. In: *BMC psychiatry* 15, S. 259. DOI: 10.1186/s12888-015-0633-4.

Giesinger, Johannes; Rumpold, Mag. Gerhard; Schüßler, Gerhard (2008): Die K10-Screening-Skala für unspezifischen psychischen Distress. In: *Psychosom Konsiliarpsychiatr* 2 (2), S. 104–111. DOI: 10.1007/s11800-008-0100-x.

Guizzaro, Lorenzo; Pétavy, Frank; Ristl, Robin; Gallo, Ciro (2021): The Use of a Variable Representing Compliance Improves Accuracy of Estimation of the Effect of Treatment Allocation Regardless of Discontinuation in Trials with Incomplete Follow-up. In: *Statistics in Biopharmaceutical Research* 13 (1), S. 119–127. DOI: 10.1080/19466315.2020.1736141.

Kessler, R. C.; Andrews, G.; Colpe, L. J.; Hiripi, E.; Mroczek, D. K.; Normand, S. L. T. et al. (2002): Short screening scales to monitor population prevalences and trends in non-specific psychological distress. In: *Psychological medicine* 32 (6), S. 959–976. DOI: 10.1017/s0033291702006074.

Kessler, Ronald C. (2001): Kessler Psychological Distress Scale (K10). Online verfügbar unter https://www.tac.vic.gov.au/files-to-move/media/upload/k10_english.pdf.

Polverejan, Elena; Dragalin, Vladimir (2020): Aligning Treatment Policy Estimands and Estimators—A Simulation Study in Alzheimer’s Disease. In: *Statistics in Biopharmaceutical Research* 12 (2), S. 142–154. DOI: 10.1080/19466315.2019.1689845.

Rubin, Donald B. (1987): Multiple Imputation for Nonresponse in Surveys. Hoboken, NJ, USA: John Wiley & Sons, Inc.

Schulz, Kenneth F.; Altman, Douglas G.; Moher, David (2010): CONSORT 2010 Statement: updated guidelines for reporting parallel group randomised trials. In: *BMC medicine* 8, S. 18. DOI: 10.1186/1741-7015-8-18.

Note to File: Facing Depression Together (Gemeinsam durch die Depression/WESPA)

DRKS00025241

This Note to File describes issues with the statistical evaluation of the „Facing Depression Together“ study along with measures taken.

Item	Description of issue and measures taken
Planned analysis by gender<br>Table A4-17	In the analysis of K-10, the subgroup of diverse gender was omitted due to data protection issues: There were only two caregivers of diverse gender, one in the AUT and TAU arm, respectively.
Tables A1-1, A1-2, A1-4	Wrong numbers/percentages of all caregivers who gave informed consent (Total column) and of screening failures are displayed in Tables A1-1, A1-2, A1-4. The correct numbers are shown in the CONSORT diagram (Figure B1-2). Numbers and percentages per randomized arm are correct. A comment was added to the tables.
Table A1-6	In the first line (Informed consent of depressed persons by randomized caregiver), the total numbers and percentages are incorrect. Numbers and percentages per randomized arm and the lines that follow are correct. A comment was added to the table.
Planned Table A6-2, serious adverse events	All adverse events were assessed by several members of the study team as either serious or non-serious according to the GCP (Good Clinical Practice) criteria. Eleven adverse events were found to be serious, and a description was included in the study report. This was done after the data base lock. The corresponding tables were not produced.
